# Psychophysical Tests Do Not Identify Ocular Dominance Consistently

**DOI:** 10.1177/2041669519841397

**Published:** 2019-04-29

**Authors:** Miguel A. García-Pérez, Eli Peli

**Affiliations:** Departamento de Metodología, Facultad de Psicología, Universidad Complutense, Madrid, Spain; The Schepens Eye Research Institute, Massachusetts Eye and Ear, Department of Ophthalmology, Harvard Medical School, Boston, MA, USA

**Keywords:** binocular vision, ocular dominance, psychophysical tests, validity

## Abstract

Classical sighting or sensory tests are used in clinical practice to identify the dominant eye. Several psychophysical tests were recently proposed to quantify the magnitude of dominance but whether their results agree was never investigated. We addressed this question for the two most common psychophysical tests: The *perceived-phase test*, which measures the cyclopean appearance of dichoptically presented sinusoids of different phase, and the *coherence-threshold test*, which measures interocular differences in motion perception when signal and noise stimuli are presented dichoptically. We also checked for agreement with three classical tests (Worth 4-dot, Randot suppression, and Bagolini lenses). Psychophysical tests were administered in their conventional form and also using more dependable psychophysical methods. The results showed weak correlations between psychophysical measures of strength of dominance with inconsistent identification of the dominant eye across tests: Agreement on left-eye dominance, right-eye dominance, or nondominance by both tests occurred only for 11 of 40 observers (27.5%); the remaining 29 observers were classified differently by each test, including 14 cases (35%) of opposite classification (left-eye dominance by one test and right-eye dominance by the other). Classical tests also yielded conflicting results that did not agree well with classification based on psychophysical tests. The results are discussed in the context of determination of ocular dominance for clinical decisions.

## Introduction

Ocular dominance has received attention in vision science for decades, but its determination started to be relevant for monovision contact lens correction of presbyopia (where one eye is corrected for distance viewing and the other is corrected for near vision; Evans, 2007), and it may be relevant also for fitting peripheral prisms for field expansion in homonymous hemianopia (Ross, Bowers, & Peli, 2012). Determination of ocular dominance is even more relevant for less reversible procedures involving monovision laser refractive surgery and intraocular lens implantation. The general practice in monovision correction is to correct the dominant eye for distance (Harris & Classé, 1988; Jain, Arora, & Azar, 1996; Raju, 2016), as blur suppression is supposedly easier in the nondominant eye and near vision typically involves high-contrast conditions (e.g., reading) in which even the nondominant eye should do well (Finkelman, Ng, & Barrett, 2009). The notion that the dominant eye has better vision is inherent to this stance, but empirical evidence speaks against this notion (Pointer, 2007).

Many classical tests of ocular dominance exist, but their results are contradictory. Coren and Kaplan (1973) administered the 13 tests most frequently used at the time to 57 normally sighted observers, and they found a diverse pattern of correlations. Factor analysis identified three groups of tests regarded as measuring what they called sighting, sensory, or acuity dominance (see also Porac & Coren, 1976; for a similar study, see Gronwall & Sampson, 1971). It is still unclear whether the tests in each group isolate different aspects of a multifaceted construct or, rather, they measure ocular dominance in combination with procedural characteristics of the task that each test poses (see Pointer, 2010a, 2012). On the other hand, it is clear that acuity tests define dominance as better monocular vision. These difficulties explain the interest in distinguishing ocular dominance from ocular preference (Laby & Kirschen, 2011; Pointer, 2010b) and the shift of perspective from defining the dominant eye as that used in monocular tasks to defining dominance according to each eye’s contribution to cooperative–competitive processes elicited during binocular vision (Han, He, & Ooi, 2018; Johansson, Seimyr, & Pansell, 2015; Mapp, Ono, & Barbeito, 2003). It is also noteworthy that classical tests of ocular dominance measure it as a dichotomous variable (either left-eye [LE] dominance or right-eye [RE] dominance, perhaps also with the intermediate outcome of nondominance). Tests that measure quantitatively the degree of ocular dominance thus seem necessary under the new perspective. See Haun and Peli (2014) for some practical implications of different monocular contributions to vision.

Several psychophysical tests were introduced in the late 2000s to measure ocular dominance quantitatively. One of them, proposed by Huang, Zhou, Lu, Feng, and Zhou (2009), uses the *interocular combination paradigm* that J. Ding and Sperling (2006) devised to investigate contrast gain control between the eyes. The test uses a spatially superimposed dichoptic display of sinusoids of opposite phases that also differ in contrast. By measuring how the perceived phase of the cyclopean percept varies as a function of the contrast ratio of the sinusoids, the test aims to estimate the ratio at which both eyes contribute equally to the cyclopean combination. A second test, proposed by Li et al. (2010), uses instead the *interocular interference paradigm* devised by Hess, Hutchinson, Ledgeway, and Mansouri (2007) and Mansouri, Thompson, and Hess (2008) to investigate binocular interactions. Here, one eye sees a field of randomly located signal dots moving in a common direction, while the other eye sees a spatially superimposed field of noise dots moving in random directions. The test estimates the percentage of signal dots (called coherence threshold) needed for identification of direction of motion with each eye; the dominant eye is taken to be that with the lower threshold, and the magnitude of dominance is defined as the ratio of thresholds in the nondominant and dominant eyes. A third test, proposed by Yang, Blake, and McDonald (2010), uses an *interocular suppression paradigm* that presents to an eye a target arrow whose contrast progressively increases while that of a spatially superimposed noise Mondrian presented to the other eye progressively decreases. Observers respond when they identify the direction to which the arrow points. The time of the response is a proxy to the threshold contrast for detection in one eye with noise coming from the other. This test is inconclusive when the eyes have different contrast sensitivities, and it will not be considered in this article. Other tests also not considered in this article are based on *binocular rivalry paradigms* of various sorts (e.g., Dieter, Sy, & Blake, 2017; Y. Ding, Naber, Gayet, Van der Stigchel, & Paffen, 2018; Handa et al., 2006; Handa, Shimizu, Uozato, Shoji, & Ishikawa, 2012; Valle-Inclán, Blanco, Soto, & Leirós, 2008; Xu, He, & Ooi, 2010; see also Bossi, Hamm, Dahlmann-Noor, & Dakin, 2017).

These psychophysical tests rest on different principles, but they are all claimed to measure ocular dominance. Yet, the extent to which their outcomes agree with one another has never been investigated. The first two tests have been used in within-subject studies in two papers that addressed other issues (Z. Chen et al., 2016; Zhou, Clavagnier, & Hess, 2013), but the authors did not collect enough data for comparisons or did not present data in a way that allows comparisons. Hence, it is unknown whether ocular dominance measures obtained with these two tests (or the other psychophysical tests, for that matter) correlate any more strongly than do measures obtained with the classical tests discussed earlier. If the results of different tests did not correlate, each test would be measuring something different, and it might not even be ocular dominance. The suspicion gains support from the results of a study that used prolonged monocular adaptation to deprive one eye from phase information (Bai, Dong, He, & Bao, 2017), which revealed that the manipulation had no effect on ocular dominance measured with the perceived-phase test although it affected substantially the dynamics of binocular rivalry (and, hence, the outcome of rivalry tests of ocular dominance). This finding suggests that ocular dominance may not be the main determinant of performance in all the psychophysical tests that are claimed to measure it.

In addition, the psychophysical methods conventionally used to gather data for the two tests to be considered in this study are known to be inadvisable: The perceived-phase test of Huang et al. (2009) uses the bias-prone method of adjustment, and the coherence-threshold test of Li et al. (2010) also uses the bias-prone single-presentation method with a forced-choice response format. More accurate quantification of ocular dominance (were this what the tests measure) would be obtained if the tests were administered with dependable psychophysical methods.

The research reported in this article addressed these issues. Specifically, we studied the agreement between ocular dominance measured with the perceived-phase and the coherence-threshold tests in their original form and under variants that use advisable psychophysical methods. The study also included some of the classical tests of sensory dominance: the L + R suppression check of the Randot Stereotests, the Worth 4-dot test, and the Bagolini lenses test. It should be noted that all these tests were proposed and used without a preliminary definition of what ocular dominance is and also without a theoretical argument indicating how ocular dominance by that inexisting definition (and not something else) manifests through the task that each test poses. In a sense, all testing proceeds on the implicit assumption of an operational definition by which ocular dominance is whatever the tests measure. As discussed earlier, there are reasons to think that some of the tests (particularly the sighting tests that were not included in our study) measure ocular preference rather than dominance, and it is also not clear whether monocular suppression as found in amblyopia and other forms of abnormal binocular vision masquerades as ocular dominance in these tests or it is instead a consequence of actual ocular dominance. On the other hand, it is also true that not any outcome measure obtained from the results of a task involving the interocular combination of noncongruent monocular inputs is necessarily indicative of ocular dominance, the more so in the absence of an explicit definition of how ocular dominance operates. Our position is that ocular dominance is a nonclinical condition that manifests when the percept reported by an observer presented with noncongruent inputs to each eye reflects a stronger contribution from one of the eyes, but we should stress that the goal of this article is not to propose a definition of ocular dominance or a test that follows from that definition. Rather, our only goal is to investigate the agreement (or lack thereof) among psychophysical tests claimed to measure ocular dominance. The main sample for our study consists of 40 normally sighted individuals for whom ocular dominance could not be confounded with other factors, but we also used an incidental second sample of four patients for whom the presumed measures of ocular dominance obtained with these tests might indeed be only reflecting collateral consequences of their condition (i.e., that the two eyes are not in a fair competition).

The organization of the article is as follows. The next section describes the two psychophysical tests and the three classical tests of ocular dominance used in this study, their original form of administration, and the alternative form in which they will also be administered here. Next, we describe our methods and results, which reveal major discrepancies among tests. Specifically, classical tests did not agree with one another, not a novel result indeed. The same was true for psychophysical tests in their conventional form of administration: The perceived-phase test classified observers either as LE dominant or as nondominant, whereas the coherence-threshold test instead classified observers either as RE dominant or as nondominant, but with very few observers classified as nondominant by both tests. On the other hand, conventional and alternative forms of administration of the perceived-phase test agreed reasonably well. Yet, the alternative form of administration of the coherence-threshold test revealed major differences in motion perception between the nasal and temporal retinae of either eye. These within-eye differences precluded the identification of a dominant eye. No comparisons could thus be conducted that involved the alternative coherence-threshold test, but identification of major within-eye differences in motion perception casts doubts on the validity of the conventional coherence-threshold test of ocular dominance.

## Tests and Forms of Administration

### Perceived-Phase Test

This test finds immediate formal justification. Suppose a sinusoid with contrast *m*_1_ and phase ϕ_1_ (in rad) is presented to one eye and an otherwise identical sinusoid with contrast *m*_2_ and phase ϕ_2_ is presented to the other eye, and recall the trigonometric identity

(1) $$a_{1}m_{1}\text{sin}(x+\phi_{1})+a_{2}m_{2}\text{sin}(x+\phi_{2})=a\text{sin}(x+\phi )$$

with

(2) $$a=\sqrt{a_{1}^{2}m_{1}^{2}+a_{2}^{2}m_{2}^{2}+2a_{1}m_{1}a_{2}m_{2}\text{cos}(\phi_{2}-\phi_{1})}$$

(3) $$\phi =\phi_{1}+\text{tan}-1(\frac{a_{2}m_{2}\text{sin}(\phi_{2}-\phi_{1})}{a_{1}m_{1}+a_{2}m_{2}\text{cos}(\phi_{2}-\phi_{1})})$$

**Figure 1.**
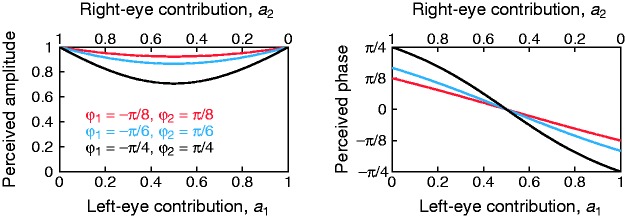
Theoretical perceived amplitude and phase of the cyclopean combination of sinusoids with several interocular phase offsets ϕ_1_ and ϕ_2_ (see the legend in the left panel) as a function of the contributions of the eyes to the cyclopean percept.

Although the two monocular images are obviously not immediately added algebraically before further processing, the right-hand side of Equation (1) captures phenomenologically the cyclopean image arising from separate stimulation of each eye with one of the sinusoids on the left-hand side, where *a*_1_ and *a*_2_ (with 0 ≤ *a*_1_,*a*_2_ ≤ 1 and *a*_1_ + *a*_2_ = 1) are ocular contributions to the binocular percept. By representing ocular contributions, *a*_1_ and *a*_2_ may also capture the effects of interocular gain control mechanisms when *m*_1_ ≠ *m*_2_, and this is perhaps the reason that J. Ding and Sperling (2006) implicitly assumed no ocular dominance, for otherwise contrast gain control would have been undecipherable from the data. The implicit assumption manifests in that their account of data adheres to Equation (3) with *a*_1_ = *a*_2_ = 0.5 (i.e., no ocular dominance) when *m*_1_ = *m*_2_. Equations (2) and (3) describe how the perceived amplitude and phase of the cyclopean percept vary with ocular weights and stimulus contrasts and phases. These relations are illustrated in Figure 1 for sample pairs (ϕ_1_, ϕ_2_) when *m*_1_ = *m*_2_ so that gain control is not confounded with ocular weights.

Empirical applications of this test (e.g., Feng et al., 2017; Zhou, Feng, Lin, & Hess, 2016; Zhou, Wang, Feng, Wang, & Hess, 2017) typically use a large fixed contrast *m*_2_ for the sinusoid presented to the eye previously determined to be nondominant, with ϕ_1_ and ϕ_2_ also fixed at opposite values. Then, perceived phase ϕ is measured at a number of contrast ratios δ = *m*_1_/*m*_2_. A curve fitted to the data estimates the *balance point*, defined as the contrast ratio δ at which ϕ = 0. Strength of dominance is then indicated by how low δ is at the balance point. Yet, due to its theoretical foundations, this test can provide a direct estimate of the ocular weights *a*_1_ and *a*_2_ from the perceived phase ϕ at any contrast ratio δ, something that was not noted by its proponents. Indeed, algebraic manipulation of Equation (3) yields

(4) $$a_{1}=\frac{1}{1-\delta \csc(\phi -\phi_{2})\text{sin}(\phi -\phi_{1})}$$

(5) $$a_{2}=1-a_{1}=\frac{\delta }{\delta -\csc(\phi -\phi_{1})\text{sin}(\phi -\phi_{2})}$$

It is also apparent that ocular weights determined via Equations (4) and (5) are invariant across changes in ϕ_1_ and ϕ_2_. Also, if δ = 1 to eliminate the influence of contrast gain control, *a*_1_ and *a*_2_ are indicative of ocular contributions to the binocular combination and, hence, of ocular dominance. In contrast, the balance point estimated as described earlier varies with ϕ_1_ and ϕ_2_, and it includes an inevitable component from contrast gain control. In other words, the balance point is only an indirect and contaminated ordinal index of ocular dominance.

In practice, and following J. Ding and Sperling (2006), perceived phase ϕ is measured in this test by displaying a thin dark line oriented along the stripes of the sinusoid and asking observers to adjust its position in the orthogonal dimension until it coincides with the perceived center of a dark stripe in the cyclopean percept. This is the conventional form of administration that will be used in this study. Yet, other forms have been devised using also the method of adjustment, but now to match the contrast and phase of a monocularly presented sinusoid to the perceived contrast and phase of the cyclopean percept (Huang, Zhou, Lu, & Zhou, 2011; Huang, Zhou, Zhou, & Lu, 2010).

Our alternative form of administration uses a dual-presentation paradigm to separate sensory from decisional components of performance (García-Pérez & Alcalá-Quintana, 2013) and which was shown in other contexts to be immune to the problems associated with the use of the method of adjustment (see García-Pérez & Peli, 2014). In this paradigm, standard sinusoids for cyclopean combination are briefly presented next to an also dichoptic display of a probe sinusoid whose phase is common to both eye’s view so that, for all practical purposes, the probe is seen binocularly. (This is also captured by Equations (1)–(3) when *m*_1_ = *m*_2_ and ϕ_1_ = ϕ_2_, regardless of the values that *a*_1_ and *a*_2_ might have.) Observers then report whether both sinusoids appear to have the same phase. With repeated presentation of several probe phases and with position (left or right of the fixation point) balanced across trials, psychometric functions for “same” responses can be obtained to estimate the point of subjective equality (PSE), that is, the probe phase that is perceptually equal to the phase of the cyclopean combination. With a suitable analysis, the same–different paradigm with dual presentations isolates the sensory component of the PSE (see García-Pérez & Alcalá-Quintana, 2017) and, hence, provides a dependable estimate of the perceived phase ϕ from which ocular weights can be obtained via Equations (4) and (5).

In addition, and compared with prolonged viewing of the sinusoid in the conventional form of administration, brief exposure in the dual-presentation variant reduces desensitization or afterimages substantially and virtually eliminates the instability arising from rivalry processes.

### Coherence-Threshold Test

The coherence-threshold test lacks formal foundations and apparently rests only on the traditional and questionable notion that the dominant eye is that which is better at some task. Each trial in the test presents a spatial field with a fixed number of dots some of which (signal dots) move coherently to the left or to the right and are seen with one eye, whereas the remainder (noise) dots are seeing with the other eye and each moves in a random direction. Multiple trials are administered to estimate the percentage of coherently moving dots needed to achieve a threshold performance level, defined as some probability of correct identification of direction of motion. Interwoven series of trials estimate the coherence threshold in each eye, and these thresholds may obviously differ between eyes, but no formal argument suggests that these differences reflect ocular dominance or how. The test seems to rely on the assumption that motion signals extracted separately from each eye compete to produce a sensation of coherent motion of certain strength. Thus, coherence thresholds will be lower when signal dots are presented to the eye that either produces a stronger coherent-motion signal or that has a stronger contribution to the overall sensation of coherent motion. The adequacy of this justification requires that coherence thresholds do not differ between eyes in monocular conditions (i.e., when signal and noise dots are both presented to only one eye) because dichoptic thresholds would be otherwise uninterpretable. Monocular thresholds are never measured in the application of this test, but the ratio of dichoptic thresholds between the eyes is taken as a measure of ocular dominance.

One might surmise that the lack of a formal argument supporting this test was expiated by empirical evidence of its validity, but all the relevant evidence reported in the seminal papers suggested instead that this test does not measure ocular dominance in normally sighted observers. With eight observers, Mansouri et al. (2008; see their Figure 3) did not find differences between average coherence thresholds in dominant and nondominant eyes determined by a sighting test. Interestingly, monocular thresholds also did not differ meaningfully between eyes. Earlier, Hess et al. (2007) had reported similar evidence that this test is insensitive to interocular interactions, although their study reported only essentially identical monocular and dichoptic thresholds averaged across the eyes of each of three observers. Also, no evidence of validity was provided by Li et al. (2010), and they did not measure monocular thresholds for comparison. Nonetheless, the test does appear to differentiate amblyopes from normally sighted observers (Black, Thompson, Maehara, & Hess, 2011; Li et al., 2011; Mansouri et al., 2008). Then, this test definitely identifies suppression in amblyopes, but it does not seem to identify dominance in normally sighted observers.

Besides the lack of a formal link with ocular dominance and the negative evidence of validity, three suboptimal features meet in the conventional form of administration of this test. One is that it uses the single-presentation method whereby observers report a categorical judgment for the cyclopean combination that they perceive in each trial and, thus, an unknown internal criterion determines the response. Another feature is that the judgment must be reported as motion to the left or to the right, forcing observers to respond at random in the surely many trials in which all motion appears to be in disparate directions. Single-presentation methods with instructions that force observers to give random responses are a source of bias and wild estimates (García-Pérez & Alcalá-Quintana, 2013). The third feature is that thresholds are estimated via average of reversals in up–down staircases, a method known to give unpredictably disparate outcomes (García-Pérez, 1998, 2000, 2011). Our implementation of this test will include the two former features (single-presentation form and binary forced choice), but we will replace the rowdy threshold estimation procedure with a more dependable one based on fitting psychometric functions.

Our alternative implementation follows from the same considerations motivating our variant of the perceived-phase test. A dual-presentation method is used to determine thresholds in each eye using a ternary response format in which observers report indecision instead of responding at random, as in García-Pérez and Peli (2015). In such a variant, a partly coherent motion field analogous to that used in the conventional test is presented side by side with an incoherent field in which all dots move in random directions, with the position of each field (left or right of the fixation point) balanced across trials. An additional advantage of this variant is that the coherently moving dots are systematically presented either to the nasal or to the temporal retina of either eye, allowing for a control of well-known nasotemporal asymmetries (see, e.g., Fahle, 1987) whose confounding effects are inextricably mixed up in the conventional form of administration of the test.

### Classical Tests

Three classical sensory tests were also used in this study in conventional and alternative forms. In the Worth 4-dot test, observers wear green and red filters before the LE and the RE, respectively, and look at an instrument that displays two green dots, a red dot, and a white dot. Ocular dominance is conventionally inferred from the perceived color of the white dot: Red indicates RE dominance, green indicates LE dominance, and alternation, mixture, or combination (yellow) of red and green indicates no dominance. We administered the test a second time after swapping the color filters between the eyes.

In the Bagolini test, observers wear plano lenses with striations along the 45° meridian in the LE and along the 135° meridian in the RE while looking at a point light source appearing through the lenses as line streaks. Ocular dominance is inferred from the pattern reported by the observer: Absent or disappearing lines at only one of the orientations is indicative of dominance of the other eye, whereas line presence or disappearance at both orientations is interpreted as lack of ocular dominance. We also administered this test after swapping the lenses between the eyes.

In the L + R suppression check of the Randot Stereotests, where observers look at stereograms through polarized glasses, letter L and the horizontal segment of the plus sign are visible to the LE, whereas letter R and the vertical segment are visible to the RE. Ocular dominance is inferred by the observer’s description of the perceived pattern: Whether they report L + R either constantly or with alternating disappearances (no ocular dominance) or, instead, report only the elements that are visible to one eye. We additionally administered this test by swapping the polarized lenses between the eyes.

## Methods

This research followed the tenets of the Declaration of Helsinki, and the institutional review board approved its protocol. Observers gave written informed consent prior to their participation.

### Observers

Results are reported for data from 23 male and 17 female observers. Their ages ranged from 17 to 77 years (mean: 34.3, standard deviation [SD]: 15.3), and they were refracted prior to the study. If the difference in refraction between habitual and best correction exceeded 0.37D in spherical equivalent, or if acuity differed between the eyes by more than five letters (one line in standard acuity charts), all testing was done with the best corrected prescription in a trial frame. All observers met the following eligibility criteria: (a) no strabismus; (b) normal or corrected-to-normal acuity of 20/20 or better in each eye; (c) normal stereo acuity (< 70 sec of arc) measured by the Randot Stereotest; (d) normal binocular vision (fusion) in the dark determined with the Worth 4-dot test; and (e) for male observers, normal color vision in each eye measured by the Ishihara test. Only one candidate observer was found ineligible and did not participate in the study. An eligible observer withdrew halfway through the study, and his incomplete data were discarded. All observers except one of the authors were naïve as to the goals of the study.

### Apparatus

In all psychophysical tests, stimuli were displayed on two 42-inch VO42L FHDTV10A monitors (VIZIO Inc., Irvine, CA, USA) facing each other at a distance of 125 cm for viewing through a mirror stereoscope placed between them (for a full description of the device, see Hwang, Deng, Gao, & Peli, 2017). The frame rate was 60 Hz, and the monitors were gamma corrected. Monitors had a resolution of 1,920 × 1,080 pixels, and their display area was 93 × 52 cm. Observers wore a Model #51 side-shielded fit-over frame with no lenses (NoIR LaserShields, South Lyon, MI, USA) to block the actual stimuli that might be visible in their outermost periphery. A chin rest ensured an effective viewing distance of 62.5 cm so that the image area subtended approximately 73 × 45°. Stimulus presentation and response collection were controlled by a computer running custom MATLAB scripts that called Psychtoolbox-3 (http://psychtoolbox.org) functions.

### Stimuli

**Figure 2.**
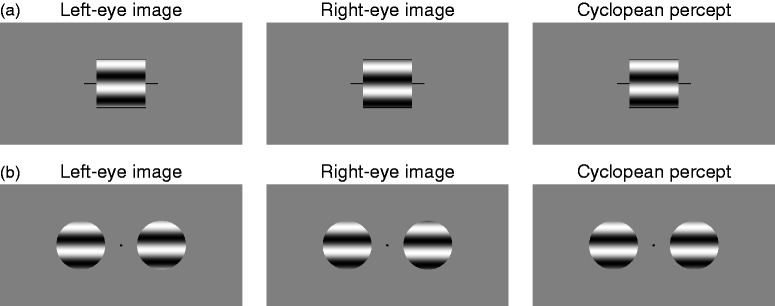
(a) Stimuli for the conventional administration of the perceived-phase test. In this illustration, the left-eye image (left panel) has phase ϕ_1_ = −π/8 rad (−22.5°) and the right-eye image (center panel) has phase ϕ_2_ = π/8 rad (22.5°). The phase of their combination in the cyclopean percept (right panel) depends on the magnitude of the contribution from each eye, assumed to be even in this illustration (i.e., *a*_1_ = *a*_2_ = 0.5, which makes ϕ = 0 in Equation (3)). (b) Sketch of the stimuli in the dual-presentation variant of the perceived-phase test; stimuli were Gabor patches, not the circularly-windowed gratings shown here for simplicity. The patch on the right of fixation has phase ϕ_1_ = −π/8 rad (−22.5°) in the left-eye image (left panel) and ϕ_2_ = π/8 rad (22.5°) in the right-eye image (center panel). The phase of their combination in the cyclopean percept (right panel) is also illustrated with *a*_1_ = *a*_2_ = 0.5 so that ϕ = 0 in Equation (3). The left-eye and right-eye patches on the left of fixation are shown in this illustration with ϕ_probe_ = 0. *Note:* Reproduction of the sinusoids may have artifactual horizontal lines not present in the actual stimuli.

Stimuli differed across tests and formats of administration. For the conventional perceived-phase test, two cycles of a horizontal 0.2 c/deg grating were created in a 240 × 240-pixel array that subtended ∼10°. Stimuli were displayed at 25% contrast, and one-pixel-wide horizontal black lines were added at their top and bottom as fusion locks. One-pixel-wide horizontal line segments stuck out on the left and right of stimuli; these segments moved synchronously up or down at the observer’s command during the adjustment task, and they also served as fusion locks. Stimuli appeared on a uniform gray background of 60 cd/m^2^ that covered the image area. Two versions of the grating were created with ϕ_1_ = −π/8 rad and ϕ_2_ = π/8 rad (i.e., ±22.5°) for dichoptic presentation (see Figure 2(a)). The phase shift is 0.625° of visual angle (1.09 prism diopters) and the patterns should thus be fusible by observers with normal binocular vision. A zero-phase version was also created for its dichoptic presentation to equate direct binocular viewing. Note in Figure 2(a) that a grating with zero phase is in sine phase so that the center of its upper dark stripe is above the vertical center of the square window. This prevented observers from transforming the task into (or supplementing it with) bisection of the vertical length of the window.

**Figure 3.**
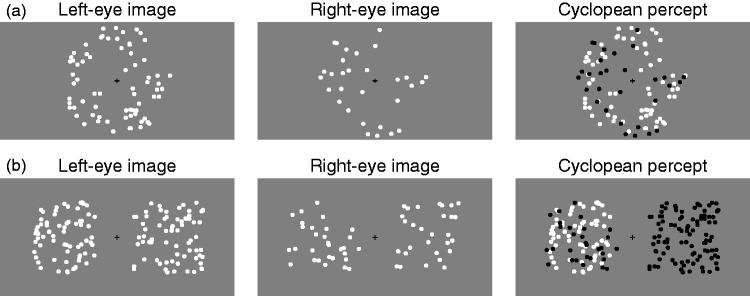
(a) Stimuli for the conventional administration of the coherence-threshold test, not to scale. In this illustration, the left-eye image contains 70 dots moving, for example, to the right, whereas the right-eye image contains 30 dots each moving in a random direction beyond ±30° from rightward motion. Combination in the cyclopean percept results in dots moving coherently (depicted in white here) amid dots moving in random directions (depicted in black here). Coherent motion could be leftward or rightward and could be presented to the LE or to the RE. (b) Stimuli in the dual-presentation variant of the coherence-threshold test, with the same graphical conventions and also not to scale. Two fields of moving dots are presented side by side. One of them is thoroughly analogous to the field in the conventional test except that coherent motion is always upward; all dots in the other field move in random directions. The field with coherent motion could be presented left or right of the fixation point and to the LE or to the RE. In this illustration, dot polarity is also used in the cyclopean percept to distinguish the dots that move coherently upward (depicted in white) from those that move in random directions (in black).

Stimuli for the dual-presentation version of the perceived-phase test were 40%-contrast Gabor patches with a vertical carrier (i.e., horizontal stripes) of 0.2 c/deg. They were each created within a 300 × 300-pixel array. The space constant of the circular Gaussian envelope was 0.65 carrier cycles (3.25°). Two patches were displayed on each monitor with 320 pixels (∼12°) of separation between their centers (see Figure 2(b)). The LE and RE components of one of them had phases ϕ_1_ = −π/8 rad and ϕ_2_ = π/8 rad, respectively, making up what we will refer to as the standard stimulus. The LE and RE components of the other (probe) patch had a common phase ϕ_probe_ that varied across trials as determined by the procedure (see later). A small black fixation dot was continuously present between the two stimuli as a fusion lock.

The stimulus for the conventional coherence-threshold test was a field of 100 moving dots split into two groups for dichoptic presentation. The size of each group varied as determined by the procedure (see later). The dots in one group moved coherently to the left or to the right; the dots in the other group moved each in a random direction not within 30° of the direction of motion of the coherent field so that none of the noise dots became signal by chance. Each group was presented to one of the eyes (see Figure 3(a)). Dots were white (luminance modulation: 0.33) and 0.5° in diameter, moved at 6 deg/s, and were displayed over the gray background (60 cd/m^2^) within an unmarked ring with inner and outer radii of 2° and 10°, respectively. Dots exiting the ring were repositioned to initiate motion in the same direction at a random location in the ring. The center of the ring displayed a small black fixation cross as a fusion lock.

Stimuli for our alternative coherence-threshold test comprised two dot fields presented with a horizontal separation of 380 pixels (∼15°) between their centers (see Figure 3(b)). A small black fixation cross midway between the fields served as a fusion lock. One of the fields (the target) was analogous to that described earlier except that coherent motion was always upward, the region where the dots moved was square with a side of 10°, and dot diameter was 0.4°. The target field included dots moving coherently and presented to one eye along with dots presented to the other eye and each moving in a random direction. The other (null) field consisted of 100 dots identically split into two groups for presentation to each eye, but all dots moved in random directions within the angular constraint. The target field could appear on the right or on the left of fixation, with coherently moving dots presented to the LE or the RE.

### Procedure

An eye exam to determine eligibility was conducted first and included the administration of classical tests of ocular dominance: the L + R suppression check of the Randot Stereotests, the perceived color of the white dot in the Worth 4-dot test at near in the dark, and the Bagolini test under normal office lighting. All tests were repeated after reversing the role of the eyes, that is, swapping the Bagolini lenses, the color filters, or the polarized lenses between the eyes. Criteria for the classification of observers as LE dominant, RE dominant, or nondominant were given earlier in the description of these tests.

Psychophysical tests were then administered in a balanced Latin square to guard against order effects. Upon arrival, each observer was randomly assigned to one of the four sequences with the constraint that any sequence is administered at most once more than any other sequence at any time. Before each test, observers received instructions and completed practice sessions of the necessary length to ensure familiarity with the task and the response interface. Throughout each test, a brief textual reminder of the task in each trial and the designated response keys was present in the upper part of the display (well away from the stimulus area), which actually served as the most effective fusion lock. Specific details of the administration of each test follow.

The conventional perceived-phase test comprised eight trials for each of three conditions. In one condition (interocular offset), gratings with opposite phases were delivered to the LE and the RE, respectively; in another condition (reversed interocular offset), phase signs were reversed; in the third (control) condition, both eyes saw a zero-phase grating to mimic direct binocular vision. Each set of three consecutive trials included one trial for each condition, in the random order determined at the beginning of the session. In each trial, the line segments with which observers made their setting (see Figure 2(a)) were presented at a random vertical position between 8 and 20 pixels away in either direction from the expected setting when *a*_1_ = *a*_2_ = 0.5. Observers had unlimited time to move line segments up and down using the arrow keys of a numeric keypad until they judged it to be at the center of the upper dark stripe and then pressed the “0” key to record their setting. Movement occurred in one-pixel steps, equivalent to a change of π/60 rad (3°) in phase angle. The stimulus was removed immediately after observers entered their setting, and an intertrial interval of random length between 3 and 4 s ensured that any afterimage had decayed before presentation of the next stimulus.

Our dual-presentation version of the perceived-phase test measures the psychometric function for “same” responses with the stimuli in Figure 2(b). Data were collected in three consecutive 120-trial sessions, with a short break between sessions. Eight 15-trial adaptive staircases that probe nonmonotonic psychometric functions (García-Pérez, 2014) governed stimulus placement in each session. The staircases ran randomly interwoven. Half of them controlled presentations in which the probe was on the left of fixation, and the other half controlled presentations in which the probe was on the right. The four staircases of each type differed only in initial probe phase: −π/4 rad in two of them and π/4 rad in the other two. Observers used a numeric keypad to report whether the stripes in the left and right images appeared to be aligned (“same” response; S) or misaligned (“different” response; D). Each D response changed probe phase by one step for the next trial along that staircase; each S response changed it by two steps. Step size was π/16 rad (11.25°) in phase angle. The direction of the change varied according to the current guesstimate of the PSE, obtained as the average probe phase ϕ_tmp_ across trials with S responses (although ϕ_tmp_ = 0 until four S responses had been given). The direction of the change after an S response was randomly inward (toward ϕ_tmp_) or outward (away from ϕ_tmp_) with equiprobability, whereas the direction of the change after a D response was always inward. This placement rule deploys trials in the region of interest and maximizes the accuracy of parameter estimates (for further details, see García-Pérez, 2014). Each trial displayed stimuli for 700 ms. Observers were asked to maintain fixation on the central dot and, to allow self-pacing, they had unlimited time to respond. They could also press a third key to ask for the trial to be repeated later if they had missed the presentation. The next trial started 1,000 ms after the observer’s response.

Our implementation of the conventional coherence-threshold test measured the psychometric function for correct responses with the stimuli in Figure 3(a). Data were collected in three consecutive 120-trial sessions with breaks between them. Twelve randomly interwoven 10-trial staircases deployed stimuli in each session. Half of the staircases governed presentations in which the coherent-motion group was delivered to the LE, and the other half governed delivery to the RE. The six staircases of each type differed only in the initial percentage of dots in the coherent-motion group: 36 in two staircases, 48 in two other, and 60 in the remaining two. Observers used a numeric keypad to report whether motion was to the right or to the left, guessing when unsure. The response was classified as correct or incorrect according to the direction of motion of the coherent group, which was set at random with equiprobability on each trial. Each correct response decreased percentage coherence by one step for the next trial along that staircase; each incorrect response increased percentage coherence by two steps. Step size was 6% coherence (6 dots). Increments and decrements did not alter the total number of dots but only the balance of coherently and randomly moving dots. This setup allows accurate estimation of the parameters of monotonic psychometric functions (García-Pérez & Alcalá-Quintana, 2005). The stimulus was displayed for 1,000 ms in each trial. Observers were asked to maintain fixation on the central cross and, to allow self-pacing, they had unlimited time to respond. They could also press a third key to ask for the trial to be repeated later if they had missed the presentation. The next trial started 1,000 ms after the observer’s response.

In our variant of the coherence-threshold test, two fields with 100 dots each (see Figure 3(b)) were simultaneously displayed for 1,000 ms in each trial. Observers had to report which field (left or right of fixation) displayed dots moving upward, but they were asked to refrain from guessing and to use instead a third key to express that they could not tell, if that was the case. Observers could also press another key to ask for the trial to be repeated later if they had missed it. Fixation was required, and observers had unlimited time to respond. In each of four consecutive sessions, data were collected with 12 randomly interwoven 12-trial staircases, half of which gathered data for estimation of the LE threshold (when the target was presented to the LE), while the other half gathered data for the RE. The six staircases of each type comprised two sets that governed target presentations left or right of fixation, respectively. The three staircases in each set differed only as to the initial percentage of coherence: 36, 48, and 60. Step size was 6% coherence, and the placement rule decreased coherence by one step after each correct response (i.e., reporting the location where the target had been presented) and increased coherence by two steps after incorrect or “can’t tell” responses. The next trial started 1,000 ms after the observer’s response.

When data from all these tests had been analyzed, selected observers were asked to provide additional data on two extra conditions that represented only slight variants of the alternative perceived-phase test and the conventional coherence-threshold test. These variants were motivated by our initial results and were designed to test specific hypothesis with independent data. The variant of the alternative perceived-phase test used a standard with ϕ_1_ = ϕ_2_ = 0 rad to create a purely binocular phase-discrimination task without interocular discrepancies. The variant of the conventional coherence-threshold test delivered noise and target dots to the same eye while leaving the other eye unstimulated to create a purely monocular task for the measurement of coherence thresholds without interocular interactions.

### Data Preprocessing and Analysis

For comparison with the results of perceived-phase and coherence-threshold tests, the six classical measures of ocular dominance (three tests in two forms each) were aggregated into a global measure for each observer. This was done because two of these three tests concurred in classifying the vast majority of observer as nondominant under either form of administration (see the Results section), and, hence, separate comparison of each classical test with the psychophysical tests was meaningless. Following Li et al. (2010), we computed an average classical dominance score with the results of each individual test coded as −1 (LE dominance), 0 (no dominance), or 1 (RE dominance). Note that these scores represent only magnitude and direction of agreement among classical tests, as an average score of −1 (or 1) cannot be taken to imply complete LE (or RE) dominance.

Quantitative measures of ocular dominance were obtained from each psychophysical test as follows. In the conventional perceived-phase test, each observers’ setting is a location *y* relative to the vertical center of the grating, in pixels. The phase ϕ of a grating whose dark stripe peaks at *y* is the solution of sin(2πy/T+ϕ)=-1, where *T* = 120 pixels is the period of the grating. Then, the estimate of perceived phase is ϕ=-π(0.5+2y/T), expressed in the range (−π, π]. The final measures of perceived phase for each observer were the averages across the eight trials in each condition. Ocular weights for each observer were subsequently estimated by applying Equations (4) and (5) to half the difference between the average perceived phase in the two interocular offset conditions, a strategy presumed to remove observers’ bias (J. Ding & Sperling, 2006).

Obtaining measures in the remaining tests required fitting psychometric functions. Although the various sessions for each test ran consecutively and followed preliminary practice, we checked for stability of performance across sessions with the nonparametric test of equality developed by García-Pérez and Núñez-Antón (2018) using the generalized Berry–Mielke statistic and the software accompanying that paper. Eight tests were conducted per observer: two for the psychometric functions in the alternative perceived-phase test (one for each position of the probe), two more for the psychometric functions in the conventional coherence-threshold test (one for each eye), and four for the psychometric functions in the alternative coherence-threshold test (one for target presentations in each hemiretina of each eye). Each test checked for equality across the three or four sessions in which data were collected for the corresponding psychometric function. Thus, 8 × 40 = 320 tests were conducted, 5% of which should be significant at α = .05 when the null hypothesis of equality holds. The actual number of significant tests was 26 (8.13%), not far from the expected number of rejections of a true null. This result warranted the aggregation of data across sessions for subsequent analyses.

In our alternative perceived-phase test, S and D responses from each observer were binned by probe phase and position to fit constrained psychometric functions for each spatial position in the same–different task. We used the software provided by García-Pérez and Alcalá-Quintana (2017) to fit psychometric functions (for details, see Appendix A). The estimated PSE, as an estimate of perceived phase ϕ, was then entered into Equations (4) and (5) to estimate ocular weights for each observer.

Data from the conventional coherence-threshold test were treated as usual when thresholds are estimated by fitting psychometric functions to binomial data from single-presentation tasks. Correct and incorrect responses were binned by percentage coherence separately for presentations of the target to the LE and the RE and the three-parameter logistic function

$$\Psi (x;\beta ,\theta ,\lambda )=0.5+\frac{0.5-\lambda }{1+\text{exp}[-\beta (x-\theta )]}$$

was fitted with maximum-likelihood methods separately to data from each eye. The coherence threshold α was defined as the 84%-correct point on the lapse-free psychometric function,^1^ that is, $\alpha =\Psi^{-1}(0.84;\beta ,\theta ,0)=\theta -\text{log}(0.4706)/\beta$. Ocular dominance was then measured as the LE:RE ratio of coherence thresholds, which cannot be transformed into ocular weights.

In principle, data from the alternative coherence-threshold test were meant to be analyzed with the software provided by García-Pérez and Alcalá-Quintana (2017), which fits psychometric functions jointly at each spatial position (or temporal order) in dual-presentation tasks administered with a ternary response format. The model that the software fits assumes that the sensory effects of stimuli do not differ across the two positions or orders in which they are presented. Yet, inspection of raw data clearly revealed that such an assumption does not hold here, for reasons that will be described in the Results section. Hence, the software was amended to fit instead the ternary form of the difference model with different sensitivities (see Equations (10) and (11) in García-Pérez and Alcalá-Quintana, 2011a). The amended model can also estimate thresholds defined relative to the putative 84%-correct level (for details, see Appendix A). Ocular dominance could also be measured here as the LE:RE ratio of coherence thresholds, again with no possibility of conversion into ocular weights. Yet, it will be seen later that large within-eye differences between thresholds in the temporal and in the nasal retinae preclude the computation of a threshold ratio between eyes. These results will be presented later for completeness, but, because of the difficulties just discussed, we decided against computing an ad hoc threshold ratio that would arbitrarily end up favoring one eye or the other without an actual referent.

Agreement between commensurate indices of ocular dominance (i.e., estimates of ocular weights obtained with each form of the perceived-phase test) was evaluated through the concordance correlation coefficient (L. I. Lin, 1989; L. Lin, Hedayat, Sinha, & Yang, 2002)

$$\rho_{\text{c}}=\frac{2r_{\mathrm{xy}}s_{x}s_{y}}{s_{x}^{2}+s_{y}^{2}+(\bar{X}-\bar{Y}{)}^{2}}$$

where *X* and *Y* are the two variables whose concordance is measured. (The formula uses the conventional notation for means, variances, and correlations.) Analogously, differences between commensurate indices were statistically assessed with Bradley and Blackwood’s (1989) simultaneous test for equality of means and variances, which is more efficient and robust than separate tests for means and variances with a Bonferroni correction (García-Pérez, 2013). For incommensurate comparisons, only measures of association can be (and were) computed.

Data from the extra conditions were analyzed as described earlier for their source counterparts, except that ocular dominance cannot be inferred from binocular PSEs or monocular thresholds.

## Results

### Classical Tests of Ocular Dominance

The L + R suppression check identified 37 of 40 observers (92.5%) as nondominant and two observers as RE dominant both in regular and reversed configurations; only one observer was inconsistently classified as RE dominant in regular configuration and LE dominant in reversed configuration. Nondominance was also found consistently for 38 of 40 observers (95%) with the Bagolini test in both configurations; of the two other observers, one was nondominant by the regular configuration and LE dominant in reversed configuration, whereas the other turned up RE dominant by the regular configuration and nondominant in reversed configuration. Finally, the Worth 4-dot test rendered a broader diversity of ocular dominance, also with occasional inconsistencies in regular and reversed configurations. Only 32 of 40 observers (80%) were identically classified in regular and reversed configurations: 5 (12.5%) as LE dominant, 16 (40%) as nondominant, and 11 (27.5%) as RE dominant. It is also noteworthy that four of the eight observers who were inconsistently classified across configurations appeared to be LE dominant in one of them and RE dominant in the other.

The prevalence of nondominance by these tests in our sample of normally sighted individuals is not surprising in retrospect, given that eligibility criteria required normal binocular vision and stereopsis. The Bagolini and L + R tests actually measure suppression, which our eligible observers will hardly show to any meaningful extent. Also, ocular dominance has been found to be insignificant by classical sensory tests in normally sighted, nonpresbyopic individuals (Suttle et al., 2009), whereas it is relatively prevalent in presbyopes (Lopes-Ferreira et al., 2013), who may have developed a form of spontaneous monovision to cope with their symptoms.

On the other hand, the perceived color of the white dot in the Worth 4-dot test seems more related to the notion of dominance as an imbalance in the contribution of each eye to the creation of a cyclopean percept under interocular differences in stimulation. In any case, because the Bagolini and L + R tests classified nearly all observers as nondominant, the overall classical score computed as defined earlier is actually dominated by the results of the Worth 4-dot test.

### Conventional Perceived-Phase Test

Ocular dominance or lack thereof should show in well-defined forms in our design with three conditions. Specifically, in interocular offset conditions, dominance would show in perceived phase being closer to the phase seen by the dominant eye. As a result, estimates of perceived phase would have the same magnitude (within measurement error) but different sign in the offset and reversed offset conditions. In contrast, lack of ocular dominance would show in perceived phase around zero in both conditions. On the other hand, ocular dominance should not play any role in our control condition, where the cyclopean percept does not differ from the monocular inputs and, hence, perceived phase should be zero (within error) irrespective of dominance. Departure from these forms would reveal the presence of biases or other factors contaminating data collected with the method of adjustment.

**Figure 4.**
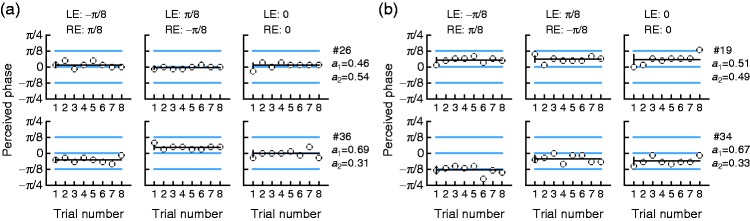
Results of the conventional perceived-phase test for sample observers in each of the three conditions (see labels at the top). Each circle in each panel is the setting in one of the trials; the horizontal line through them is the mean, and the vertical segment at the left indicates plus and minus one standard deviation. Blue horizontal lines indicate the actual phase of the components across conditions. Labels on the right show the observer number and the estimated LE and RE weights (*a*_1_ and *a*_2_). (a) Two observers whose responses match expectations based on a lack of bias and reveal negligible RE dominance (top) and moderate LE dominance (bottom). (b) Two observers whose responses violate expectations. LE = left eye; RE = right eye.

These expectations held up occasionally, as seen in Figure 4(a) for a negligibly RE-dominant observer (top) and a moderately LE-dominant observer (bottom). Yet, the most common outcome was violation of the expectation of opposite perceived phases in opposite offset conditions (see illustrative cases in Figure 4(b); results for all observers are presented in Supplementary Figure S1). Under the reasonable assumption that our control condition reveals what each observer judges to be the center of the upper dark stripe (a response criterion indeed), nearly identical results in all conditions implies that observers must be perceiving the stimulus in each of the two interocular offset conditions just as they perceive the binocular stimulus in the control condition. Thus, these observers cannot be reasonably claimed to display ocular dominance despite the nonzero phases perceived (rather, reported) in the offset conditions. To the authors’ knowledge, previous use of the perceived-phase test never included a control condition to determine bias although its signature was present for data at δ = 0, where only one eye was operating because the other eye saw uniform luminance. Inspection of empirical results presented graphically for this condition in previous studies reveals that bias was present (see, e.g., Figures 4 and 6 in Huang et al., 2009) sometimes with a very strong magnitude (see, e.g., Figures 2 and 4 in Zhou et al., 2017).

**Figure 5.**
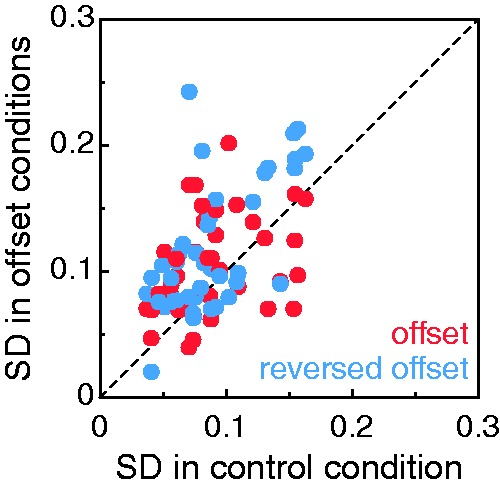
SD of the eight settings in the offset (red circles) and reversed offset (blue circles) conditions plotted against the SD of the eight settings in the control condition for each observer. The dashed diagonal is the identity line. SD = standard deviation.

**Figure 6.**
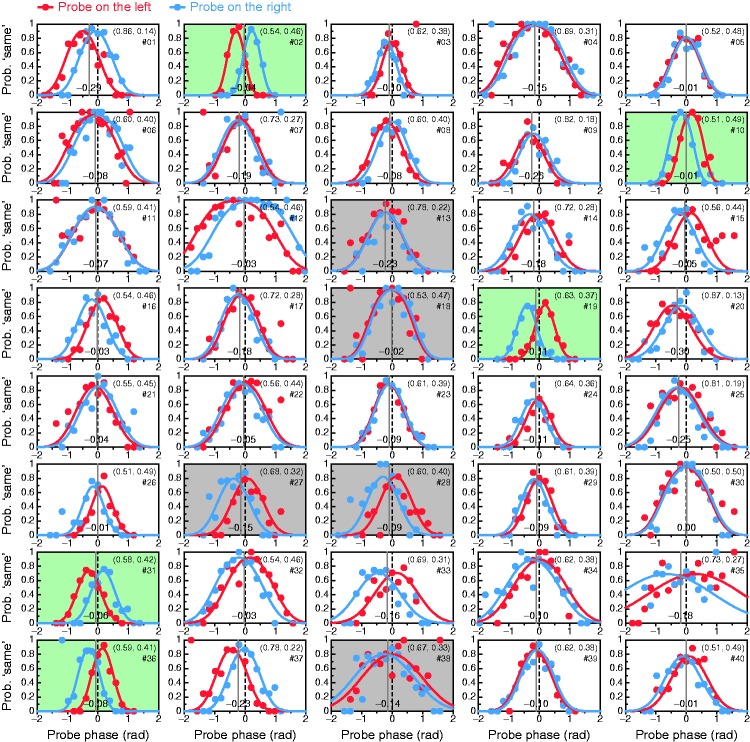
Results from the alternative perceived-phase test for all observers (panels). Psychometric functions were fitted jointly to data from trials in which the probe was on the left (red) or on the right (blue) of fixation. The dashed vertical line at zero is the expected PSE if *a*_1_ = *a*_2_ = 0.5. A continuous vertical line indicates the PSE, and the numeral on it near the bottom of the panel gives its value; estimated ocular weights (*a*_1_, *a*_2_), obtained from the estimated PSE via Equations (4) and (5), are displayed at the top right. Panels with green background denote observers producing noiseless data; panels with gray background denote observers for whom the fit was poor by the *G*^2^ statistic. Stray data points in some panels reflecting 100% “same” responses at extreme probe phases (e.g., the red data point at the rightmost probe phase in the panel for Observer #3) are likely to reflect a lapse in the single trial that was placed there by only one of the staircases comprising the adaptive method. The same is true for analogous stray data points with a smaller ordinate also at extreme probe phases, which are based on only two or three trials instead.

To control for bias, previous use of the perceived-phase test included opposite interocular offset conditions and computed perceived phase as half the difference between the average phases in them, as we did here. Relative to the illustration in Figure 4, this approach amounts to rigidly sliding up or down the average lines in the two offset conditions until each of them is on one side of the zero ordinate at the same distance from it. This strategy masks inconsistencies between perceived phases, and the meaning of the outcome is unclear on consideration that the average line for the control condition ends up away from the zero ordinate when slid by the same amount. Pretending that these difficulties do not exist, perceived phase ϕ was estimated here as usual and, via Equations (4) and (5), this rendered the estimated ocular weights (*a*_1_, *a*_2_) displayed on the right of each observer’s panels in Figure 4 (and similarly in Supplementary Figure S1).

Before we summarize overall results for this test, recall our suspicion that prolonged viewing during the adjustment task might trigger dynamic rivalry by which the cyclopean percept varies during and across trials. If this were the case, the variability of settings across trials would be larger in the interocular offset conditions than in the control (binocular) condition where rivalry is inconsequential. In search for evidence to this effect, Figure 5 plots the SD of each observer’s eight settings in each offset condition against the SD of the eight settings in the control condition. As surmised, data points fall above the identity line more often than below it, but no further statistical analyses were conducted to assess this incidental expectation.

**Figure 7.**
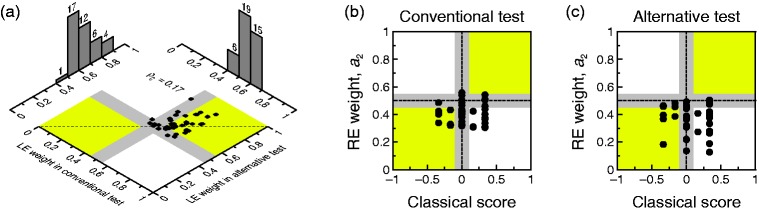
(a) Summary results for conventional and alternative perceived-phase tests. The axes of the two-dimensional plane are the estimated LE weights (*a*_1_ in Equation (1)) in each test; the dashed diagonal is the identity line. Histograms collect data into nonoverlapping bins centered from 0.05 to 0.95 in steps of 0.1. (b) RE weight estimated with the conventional perceived-phase test against average score in the classical tests. (c) RE weight estimated with the alternative perceived-phase test against average score in the classical tests. Gray stripes in each panel give reasonable ranges of nondominance along each axis. Agreement occurs if data points fall in the yellow regions or within the intersection of the gray stripes. LE = left eye; RE = right eye.

Across the board, LE weights *a*_1_ ranged from 0.44 to 0.70 with an average of 0.59 and a SD of 0.07; the frequency distribution will be displayed in Figure 7(a). Considering measurement error and meaningfulness, there is surely a region around *a*_1_ = 0.5 that reflects lack of dominance. Although the boundaries of such a region are difficult to place, we will inconsequentially assume that RE dominance can be declared if *a*_1_ < 0.45, whereas LE dominance can be declared if *a*_1_ > 0.55. It is remarkable that, by this criterion, there was a single marginal case of RE dominance (with *a*_1_ = 0.44 indeed), whereas 29 of 40 observers (72.5%) in our sample exhibited some degree of LE dominance, with *a*_1_ > 0.65 for 11 of them. Normative data were never published by the proponents of the perceived-phase test, and our results also contribute to the diverse picture arising from several other studies in which results at *m*_1_ = *m*_2_ were reported with identification of ocular dominance. For instance, the average ϕ reported by Bai et al. (2017; see the baseline condition in their Figure 5) for their eight observers was indicative of RE dominance, and most observers would surely be classified as such given the small SD. Also, Y. Chen, Wang, Shi, Wang, and Feng (2017; see their Figure 2) reported that ϕ ≈ 0 for most of their 15 normal observers, indicating nondominance; whether the remaining observers had LE or RE dominance is undecipherable. The same holds for the 40 observers in the normal control group of Kwon et al. (2014; see their Supplementary Figure S1) and for the 25 observers in the normal control group of Zhou et al. (2016; see their Supplementary Figure S3). Finally, in a recent study with a sample of 142 normally sighted observers whose LE or RE dominance was not specified, Wang et al. (2018) plotted a crude histogram of absolute values of perceived phase (see their Figure 4) that can be translated into dominance by our criterion. Specifically, by Equation (4), *a*_1_ < 0.45 or *a*_1_ > 0.55 implies meaningful dominance when the absolute-valued perceived phase is beyond 2.37°, a magnitude exceeded by about 50% of Wang et al.’s observers. Admittedly, Wang et al. did not set a criterion by which eye dominance could be claimed, and they also did not report which eye was dominant for their observers either by this test or by the sighting tests that were reportedly used for the initial classification of observers.

### Alternative Perceived-Phase Test

Our alternative test estimates perceived phase via the PSE in a dual-presentation same–different paradigm. Figure 6 shows the results for each observer. Some data sets describe very smooth paths (e.g., Observers #2, #10, #19, #31, or #36; panels with green background in Figure 6), while others are noisier. The fitted psychometric function was rejected at α = .05 by the likelihood-ratio statistic *G*^2^ for only 5 of 40 observers (#13, #18, #27, #28, and #38; panels with gray background) despite a lack of systematic deviations between fitted curves and data. The absence of systematic patterns of misfit was confirmed via unconditional residual analyses with the software provided by García-Pérez, Núñez-Antón, and Alcalá-Quintana (2015).

Psychometric functions across positions are generally displaced in opposite directions from the PSE (compare red and blue data and curves in each panel), something that is well captured by the analysis. This feature speaks again to the necessity of planning against order/position effects: Had the probe been presented at one and the same position always, only the red or the blue data points and curves would have been obtained, thus misestimating the PSE substantially in many cases. But it also speaks to the necessity of an adequate analysis: Had the data from the two presentation positions been aggregated to fit a single psychometric function, parameter estimates would also have been in error (Dyjas & Ulrich, 2014; García-Pérez & Alcalá-Quintana, 2010).

Each panel in Figure 6 shows the estimated PSE (the perceived phase ϕ) as well as the resultant estimates of ocular weights *a*_1_ and *a*_2_. Across the board, LE weights *a*_1_ ranged from 0.50 to 0.87 with an average of 0.63 and a SD of 0.10; the frequency distribution will be displayed in Figure 7(a). By the criterion used earlier, there was no case of RE dominance in our sample, whereas 30 of 40 observers (75%) exhibited some degree of LE dominance, with *a*_1_ > 0.65 for 14 of them. Conventional and alternative perceived-phase tests thus give similar results, but a more thorough comparison is worth presenting along with a comparison with the results of classical tests.

### Agreement Between Perceived-Phase Tests and Agreement With Classical Tests

Although virtually all observers were either nondominant or LE dominant by both versions of the perceived-phase test, LE ocular weights *a*_1_ estimated with our alternative test were slightly higher on average and slightly less homogeneous than those estimated with the conventional test (see Figure 7(a)). A Bradley–Blackwood test rejected equality of means and variances (*F* = 6.60, *p* = .003), and the concordance coefficient was understandably low (ρ_c_ = 0.17).

The two-dimensional plane in Figure 7(a) shows how each individual observer is classified according to each test given the nondominance boundaries defined earlier (gray shaded areas). These areas render nine regions defining a 3 × 3 contingency table, as there are three possible outcome categories (LE dominant, nondominant, RE dominant) for each test. There was a marginal case of opposite classification (*a*_1_ = 0.4407 by the conventional test and *a*_1_ = 0.5501 by the alternative test), whereas 29 of 40 observers (72.5%) were identically classified by both tests (24 as LE dominant and 5 as nondominant). We assessed categorical agreement in this two-way classification with a one-stage bootstrap test of independence that solves the problems arising from two-stage parametric tests (for details, see García-Pérez et al., 2015). At α = .05, the bootstrap test based on moment-corrected residuals did not reject independence.

Regarding the relation with classical scores, recall first that the latter capture only agreement and range from −1 (i.e., agreement on LE dominance) to 1 (agreement on RE dominance). Figure 7(b) and (c) plots the estimated RE weight *a*_2_ from the conventional and alternative perceived-phase tests, respectively, against the classical score. Categorical agreement between classical tests and perceived-phase tests as to which eye is dominant would place data points in the upper-right and lower-left quadrants (in yellow) or within the intersection of the gray stripes, something not observed here. The bootstrap test also did not reject independence in either case.

### Conventional Coherence-Threshold Test

**Figure 8.**
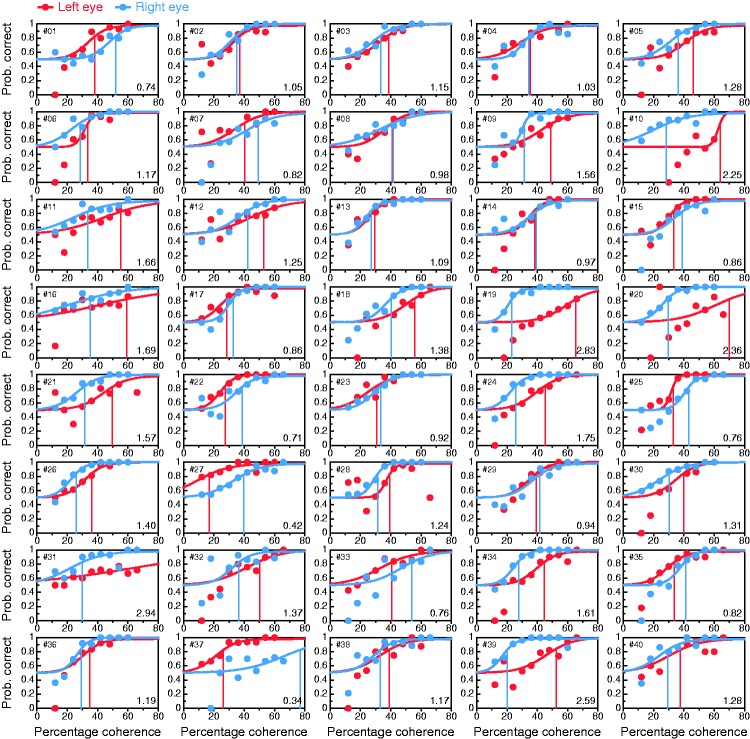
Results from the conventional coherence-threshold test for all observers (panels). Psychometric functions are fitted separately to data from trials in which the coherent group was delivered to the LE (red) or to the RE (blue), with thresholds at 84% correct indicated by vertical lines of the appropriate color. The LE:RE threshold ratio is displayed at the bottom right in each panel. As in Figure 6, occasional data points well below the expected chance level of 50% at low percentages of coherence are also here a consequence of very few trials placed by the adaptive method at those levels.

Figure 8 shows data and fitted psychometric functions for each eye in each observer. When these functions differed across eyes, the difference was in location and rarely in slope. LE:RE threshold ratios ranged from 0.34 to 2.94 with a mean of 1.3 and a SD of 0.59. Yet, appropriate treatment of threshold ratios requires a base-2 logarithmic scale, because a ratio and its inverse both reflect the same amount of dominance. Use of this transformation renders LE:RE threshold log-ratios. On this scale (where negative values indicate LE dominance and positive values indicate RE dominance), the mean threshold log-ratio was 0.24, and the SD was 0.65.

Given the measurement error in threshold estimation, declaring LE or RE dominance by placing a boundary at LE:RE = 1 seems injudicious, but it is unclear how broad a range around unity should be set to define nondominance. Previous studies do not shed any light. For instance, Li et al. (2010; see their Figure 3) found a large cluster with ratios less than 1.6 and a small second cluster with ratios greater than 1.8. Rather than understanding small ratios as a result of sampling error, they interpreted them as weak dominance to which their test was “highly sensitive,” but defining the region of nondominance as the interval [1/1.6, 1.6] is excessive. We will use Zheleznyak, Alarcon, Dieter, Tadin, and Yoon’s (2015) criterion of interocular threshold ratios within or beyond 10% of unity and will thus declare ocular dominance when the LE:RE threshold ratio is outside the interval [1/1.1, 1.1] = [0.91, 1.1]. By this criterion, only 10 of 40 observers (25%) would be declared LE dominant (i.e., LE:RE < 0.91), compared with much larger figures in the perceived-phase test (72.5% and 75%, for the conventional and alternative forms, respectively). Analogously, 23 observers (57.5%) for whom LE:RE > 1.1 would be declared RE dominant, compared with one (2.5%) and none (0%) in conventional and alternative forms of the perceived-phase test, respectively. Large discrepancies between perceived-phase and coherence-threshold tests are clearly apparent, and they will be analyzed later. Yet, our results for the coherence-threshold test are in line with those reported by Li et al. (2010): In their sample, 27 of 44 observers (61%) were RE dominant by the criterion LE:RE > 1; in our sample, 26 of 40 observers (65%) were RE dominant by the same criterion. No other study using this test seems to have reported rates of LE or RE dominance in their samples.

It is also important to stress that our finding of a relatively large number of observers with different thresholds in each eye is at odds with the original results of Hess et al. (2007) and Mansouri et al. (2008), which were discussed in the Introduction section. Our use of a dependable psychophysical method that allows estimating psychometric functions (not just thresholds) has also revealed that differences in threshold are authentic and come from psychometric functions that vary mostly in location (and rarely in slope) across eyes. Later, we will discuss how LE and RE thresholds obtained in the dichoptic conditions of this test compare with analogous thresholds obtained in the monocular scenario of one of the extra conditions defined earlier.

### Alternative Coherence-Threshold Test

**Figure 9.**
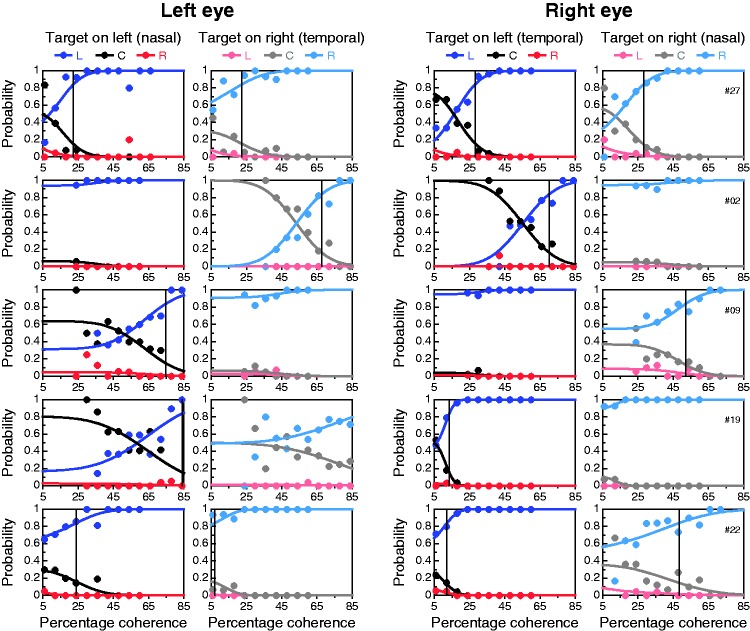
Results from the alternative coherence-threshold test for a representative observer in each of five major performance patterns (rows). Each row shows results for the LE (left side) and the RE (right side) when the coherently-moving dots were presented on the left or on the right of fixation (see labels at the top; “nasal” and “temporal” refer to the retina). Each panel shows data and psychometric functions for each possible response: upward motion on the left (L), upward motion on the right (R), or cannot tell (C). The vertical line in each panel (where shown) gives the location of estimated threshold at 84% correct; absence of the line in some panel means that the data do not provide an identifiable 84% point.

Our design of the alternative coherence-threshold test stemmed from the naïve (as it turned out) surmise that each eye can be characterized by a unique coherence threshold. Our intention was, thus, to measure the LE:RE threshold ratio with the bias-free dual-presentation method, but raw data indicated that there is not a single coherence threshold per eye. Figure 9 shows five distinct patterns that make this clear. Data and fitted functions are graphically separated by the eye to which coherent motion was delivered and by the position where the target was displayed. Data and fitted functions for all observers are presented in Supplementary Figure S2.

Observer #27 (top row), along with 20 other in our sample, shows an absence of differences in motion sensitivity across hemiretinae: The probability of a correct response (i.e., “left” when the target was on the left and “right” when it was on the right) increases with percentage coherence similarly in both hemiretinae within each eye. The four other cases illustrate different patterns of near perfect performance in some conditions and very poor and noisy performance in the others. The “nasal” Observer #2 (second row) is almost always correct even at very low percentages of coherence when the target is on the nasal retina of either eye, but performance is instead very poor in the temporal retinae; there was another observer of this type in our sample. The “temporal” Observer #9 (third row) shows the opposite pattern, and we found another observer of this type. The “right-eyed” Observer #19 (fourth row) is almost always correct even at very low percentages of coherence when the target is delivered to the RE, but performance is remarkably poorer and noisier when coherent motion is delivered instead to the LE; there were four other observers of this type in our sample plus two other observers who showed an analogous but “left-eyed” pattern. The bottom row in Figure 9 shows a final pattern characterized by very poor and noisy performance in one of the hemiretinae of one of the eyes; seven other observers showed this pattern in some form, including cases in which poor and noisy performance was observed only for target presentations on the left or on the right of fixation. Admittedly, good performance with one eye and poor performance with the other might be the result of observers’ cheating by winking one eye during stimulus presentation, but this eventuality can be ruled out. First, such strategy would not produce the relatively well-behaved data for the LE in the fourth row of Figure 9. Also, strategic cheating cannot explain nasal and temporal patterns (second and third rows of Figure 9) or the various forms of the pattern in the bottom row of Figure 9. Finally, none of the observers showing such performance admitted to cheating in any way.

**Figure 10.**
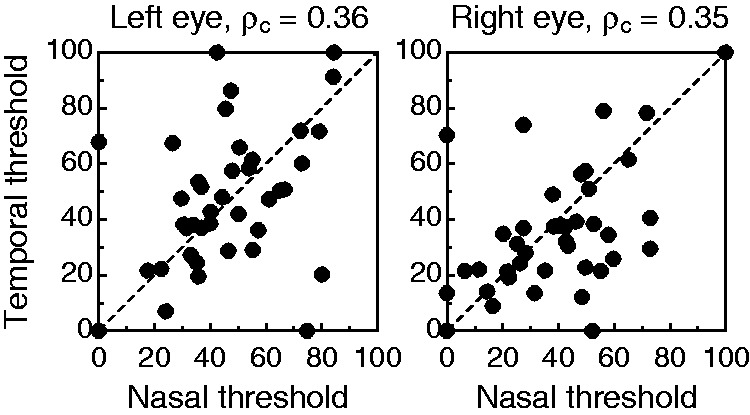
Scatter plots of temporal against nasal thresholds within the left and right eyes (panels) in the alternative coherence-threshold test. Thresholds estimated to be beyond 100% coherence due to very poor performance (see, e.g., the performance of Observer #19 in the temporal hemiretina of the LE in Figure 9) are plotted at 100%; analogously, thresholds that could not be estimated due to perfect or nearly perfect performance at the smallest percentages of coherence (see, e.g., the performance of Observer #2 in the nasal hemiretina of the LE and right eyes in Figure 9) are plotted at 0%. The dashed diagonal is the identity line, and the top shows the concordance correlation coefficient ρ_c_ between nasal and temporal thresholds, computed without the data points arbitrarily placed at 0% or 100%.

Figure 10 shows scatter plots of thresholds in the nasal and temporal retinae of each eye. Although some data points fall near the diagonal, huge scatter and low concordance can only reflect the presence of actual differences (and independence) between temporal and nasal thresholds within each eye, which poses a serious difficulty for computing LE:RE threshold ratios provided that each eye has different thresholds in the nasal and temporal retinae. That these thresholds are authentic is also corroborated by the good fit of the psychometric functions from which they are extracted (see Figure 9 and Supplementary Figure S2), another sign of the demonstrated accuracy of estimates from dual-presentation tasks with a ternary response format (see García-Pérez & Alcalá-Quintana, 2017).

Consideration of the alternative coherence-threshold test will be discontinued, but we should stress that nasotemporal asymmetries within each eye must affect in complex ways threshold ratios measured in the conventional test. Then, the conventional coherence-threshold test seems an oversimplification that may be muddying the picture of ocular interactions substantially.

### Agreement Between the Conventional Coherence-Threshold Test and the Remaining Tests

**Figure 11.**
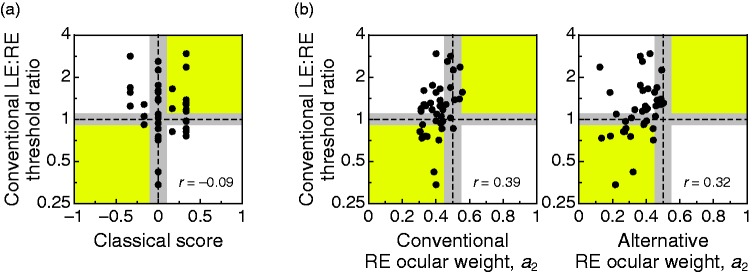
Scatter plots of LE:RE threshold ratio in the conventional coherence-threshold test against (a) average score in the classical tests and (b) RE ocular weights in the conventional (left panel) and alternative (right panel) perceived-phase tests. Product-moment correlations displayed in the insets are computed using the LE:RE threshold log-ratio. Gray horizontal and vertical stripes in each panel indicate a reasonable range of nondominance along each axis. Agreement occurs if data points fall in the yellow regions or within the intersection of the gray stripes. LE = left eye; RE = right eye.

Figure 11(a) plots the LE:RE threshold ratio from the conventional coherence-threshold test against the classical score. As before, overall agreement would place data points in the upper-right and lower-left quadrants (in yellow) and within the intersection of the gray stripes, which is not observed empirically. Also in this case, the bootstrap test of independence between classical and coherence-threshold classifications of ocular dominance did not reject the null at α = .05.

Figure 11(b) plots LE:RE threshold ratios in the conventional coherence-threshold test against RE weights estimated in each form of the perceived-phase test. Correlations are weak but positive in both cases, although positive correlation indicates agreement between tests only if the data fall in the upper-right and lower-left quadrants (in yellow) and within the intersection of the gray stripes, which is not the case. Data points lean toward the left of the plots such that observers are mostly classified as LE dominant (*a*_2_ < 0.45) by the perceived-phase test, while they are mostly classified as RE dominant (LE:RE > 1.1) by the conventional coherence-threshold test. Only 11 of 40 observers (27.5%) were equally classified by both psychophysical tests. In contrast, discrepant classification as RE dominance by the coherence-threshold test and LE dominance by the perceived-phase test was more common: 14 cases (35%) with either form of the perceived-phase test. A bootstrap test of independence based on the 3 × 3 classification did not reject the null at α = .05 for any of the two cases in Figure 11(b).

### Results in the Extra Conditions

By ensuring that the coherent group is presented either to the nasal or to the temporal retina in each eye, the dual-presentation coherence-threshold test allows investigating nasotemporal asymmetries. As it turned out, these asymmetries were huge for many observers, and their diverse forms precluded computation of LE:RE threshold ratios, questioning along the way the validity of the conventional coherence-threshold test. Indeed, a single-presentation paradigm with central fixation and dots moving coherently in the horizontal direction results in trials in which the same nominal percentage of coherence implies different strengths of motion signals according to how dots moved in each eye from the nasal to the temporal retinae (or vice versa). It is unclear how the conventional coherence-threshold test can measure ocular dominance in these conditions.

**Figure 12.**
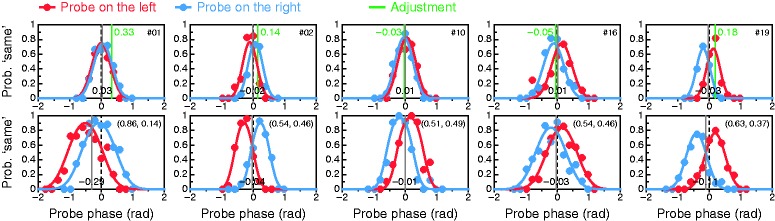
The top row shows data and psychometric functions for five observers in a binocular phase-discrimination task under conditions analogous to those in the alternative perceived-phase test, except that now both eyes see the exact same patterns. Graphical conventions as in Figure 6. The estimated PSE (perceived phase of the zero-phase standard) is marked by a solid vertical line, with its value printed near the bottom of each panel; the true value of the PSE, known to be zero in these conditions, is marked by a dashed vertical line. The green vertical line and the numeral next to it at the top of each panel is the estimate of the perceived phase of a binocularly viewed zero-phase standard obtained with the method of adjustment in the control condition of the conventional perceived-phase test. The bottom row reproduces results for the same observers from Figure 6 (alternative perceived-phase test).

The perceived-phase test is not affected by this problem: There is no motion and no distinct targets presented to the nasal or temporal retinae of one of the eyes and, more importantly, the relevant stimulus feature needed to make a judgment exists only after the cyclopean combination. Yet, we sought to determine whether some unidentified feature of interocular differences in stimulation or nasotemporal asymmetries cause the occasional lateral shifts in Figure 6, instead of these being the result of measurable decisional bias in dual-presentation paradigms. Five observers who displayed large shifts were thus asked to participate in an analogous set of sessions for the extra condition in which the components of the standard stimulus had zero phase in both eyes. This rendered a superficially identical task that differed in that vision was entirely binocular: Both eyes received the exact same stimulation in every trial, because the phase of the probe was already common to both eyes. This condition comes down to a dual-presentation phase-discrimination task with probe and standard stimuli viewed binocularly, even though through a mirror stereoscope. From Equation (3), ϕ_1_ = ϕ_2_ = 0 rad implies ϕ = 0 rad and, then, the PSE must here be at zero because probe and standard only differ along the dimension of comparison (García-Pérez & Alcalá-Quintana, 2011b). The results are shown in the top row of Figure 12, with the bottom row reproducing for comparison the results for the same observers in Figure 6.

Lateral shifts associated with probe position (left vs. right) occur in the same form in this binocular condition, revealing that the source of these shifts is unrelated to interocular differences in stimulation. Shifts are smaller here because the psychometric functions are narrower, which unsurprisingly suggests that concordant interocular input (as is found in normal binocular vision) allows more precise positional judgments. Although this extra condition ensures that the PSE is at zero, psychometric functions were fitted without imposing this constraint so as to check the accuracy of dual-presentation methods. The fact that the freely estimated PSE turns up almost at its true value of zero indicates that the small estimation error expected from simulations (see García-Pérez & Alcalá-Quintana, 2017) holds up empirically. Interestingly, this also supports the accuracy of PSEs and ocular weights estimated with our alternative perceived-phase test. In contrast, analogous estimates from the method of adjustment in the control condition of our conventional perceived-phase test (green vertical lines and numerals in each panel in the top row of Figure 12) display large departures from zero. This is another sign of bias in the method of adjustment, which surely taints estimates of perceived phase and ocular weights obtained in the conventional perceived-phase test. Appendix B presents an analysis of reliability that further confirms the superiority of our perceived-phase test over the conventional test.

**Figure 13.**
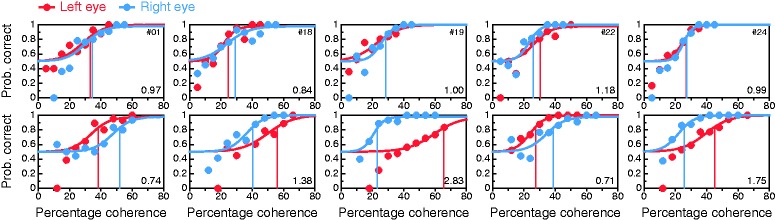
The top row shows monocular psychometric functions, thresholds, and threshold ratios for five observers in a task superficially analogous to the conventional coherence-threshold test, except that now one eye sees signal and noise dots, while the other eye sees only the fixation cross. The bottom row reproduces dichoptic psychometric functions, thresholds, and threshold ratios for the same observers from Figure 8. Graphical conventions as in Figure 8.

The second extra condition involved a task that was superficially identical to that in the conventional coherence-threshold test, except that signal and noise dots were always delivered to one of the eyes while the other eye only saw the fixation cross. The goal of this condition was to determine whether differences in threshold between the eyes are also observed when stimulation is monocular. As mentioned in the Introduction section, the two studies that motivated the development of the coherence-threshold test (Hess et al., 2007; Mansouri et al., 2008) reported indirect results indicating that monocular and dichoptic thresholds are similar in both eyes for normally sighted observers, and no other study seems to have addressed this issue. The top row of Figure 13 shows the results of monocular measurements for five observers, whereas the bottom row reproduces for comparison their dichoptic results from Figure 8. Almost invariably, monocular thresholds are meaningfully lower than their dichoptic counterparts, and they do not seem to differ much between eyes. In general, monocular psychometric functions are also steeper than their dichoptic counterparts. These results seem to suggest that interocular differences in stimulation (i.e., delivering the noise via the contralateral eye) deteriorate performance with one eye more strongly than it does with the other. Interestingly, two observers had monocular threshold ratios outside the range [0.91, 1.1] and, hence, they show signs of differences between the eyes prior to any interocular competition that might reveal ocular dominance: Monocularly, Observer #18 (second column in Figure 13) seems to have a better LE, and Observer #22 (fourth column) seems to have a better RE. Yet, and strangely enough, their dichoptic thresholds move in opposite directions: Observer #18 seems dichoptically RE dominant, and Observer #22 seems LE dominant. Anecdotal as this observation is, the fact that opposite monocular and dichoptic patterns occurred in two of the five observers that we measured can hardly be ignored.

**Figure 14.**
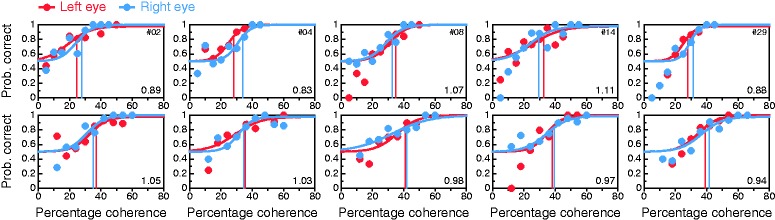
The top row shows monocular psychometric functions, thresholds, and threshold ratios for five observers whose dichoptic data (bottom row; reproduced from Figure 8) did not show differences between the eyes. Graphical conventions as in Figure 13.

The observers initially asked to serve in this extra condition were those who displayed large dichoptic differences between eyes, but the results in Figure 13 raise another question. If observers with different dichoptic psychometric functions and thresholds have similar (or reversed) monocular patterns, what are monocular functions like for observers with similar dichoptic functions? To answer this question, we sought the participation of observers whose dichoptic psychometric functions and thresholds did not differ between eyes (i.e., their LE:RE ratios were within [0.91, 1.1]). For these five observers, monocular thresholds were also lower than dichoptic thresholds (see Figure 14) and similar for both eyes although four observers (#2, #4, #14, and #29) had monocular threshold ratios outside the range [0.91, 1.1]. Monocular psychometric functions were also generally steeper than their dichoptic counterparts. Altogether, the interpretation of these results is unclear, let alone understanding how such diverse patterns of monocular and dichoptic LE:RE threshold ratios inform of ocular dominance. A casual glance at the consequences of effective suppression of one eye on performance in these tasks was taken by recruiting four observers with known binocular vision dysfunctions. The results are presented and discussed in Appendix C, and they corroborate the notion that the perceived-phase test reveals ocular contributions to binocular vision, and, hence, it is a suitable test of ocular dominance.

## Discussion

Our comparison of the outcomes of the two most widely used psychophysical tests of ocular dominance revealed very little agreement not just in the measured amount of dominance but, more dramatically, in the identification of the dominant eye. Our 40 normally sighted observers were LE dominant or nondominant by the perceived-phase test, but they were RE dominant or nondominant by the coherence-threshold test. Categorical agreement between tests only occurred for 11 of 40 observers (27.5%), whereas the dominance of 14 observers (35%) fell on opposite eyes according to each test. Both tests had been successfully used in several studies to differentiate amblyopes from normal controls, but their ability to identify suppression of one eye in amblyopia does not seem to carry over to the identification of a dominant eye in normal vision. In retrospect, it is not surprising that the poor vision in one eye of the amblyopes shows in the outcomes of the tests, which may in these and other cases of abnormal binocular vision simply indicate that the two eyes are not equally functional. In these conditions, the concept of ocular dominance as an imbalance in the contribution of two otherwise equally functional eyes to the binocular percept does not apply. Our results with normally sighted observers at least indicate that not all psychophysical tests or classical sensory tests are measuring ocular dominance by the same functional definition.

Establishing which of the two psychophysical tests (if any) measures ocular dominance by some identifiable definition is not easy without a gold standard, and the use of classical sensory tests does not seem to provide a valid criterion: Different classical tests also disagree with one another in the identification of the dominant eye, and none of them agreed reasonably well with the outcomes of either of the psychophysical tests. Nevertheless, the theoretical foundations of the perceived-phase test justify the estimation of ocular weights and make this test likely to be measuring ocular dominance as an imbalance in ocular contributions to the binocular percept. In contrast, the coherence-threshold test lacks theoretical foundations, and the diverse patterns of monocular and dichoptic psychometric functions and thresholds reported in Figures 13 and 14 undermine the interpretability of its results. These points were corroborated in our case studies involving observers with abnormal binocular vision (Appendix C). All things considered, we are inclined to think that the perceived-phase test is that which more closely comes to measuring the magnitude of ocular dominance as defined earlier. We should also stress that this potential is achieved with most precision when ocular weights are estimated as described here (not when the balance point is measured in conventional usage) and when data are collected with a dual-presentation same–different paradigm (not with the method of adjustment). Other variants of the interocular combination paradigm were recently proposed that also seek to estimate a balance point (see Bossi et al., 2017), but they require observers to report judgments of perceived contrast or direction of motion and do not produce data that can be used to estimate ocular weights.

As discussed in the Introduction section, correct identification of ocular dominance has implications for clinical decisions in which a treatment assigns different roles to each eye, as in monovision corrections (Evans, 2007) or prismatic field expansion (Apfelbaum & Peli, 2015; Peli & Jung, 2017; Ross et al., 2012). In principle, logical arguments may guide the assignment of roles, such as correcting the dominant eye for distance vision and the nondominant eye for near vision. Application of this principle presumably ensures optimal vision because blurry images coming from the near-sighted nondominant eye will be suppressed in distance vision, whereas suppression will affect instead the nonaccommodating dominant eye in near vision. However, literature reviews indicate that the success rate of monovision correction rarely exceeds 70%, most likely due to identifiable individual characteristics but surely also because suppression is not as automatic as expected (see Evans, 2007; Greenstein & Pineda, 2017; Labiris, Toli, Perente, Ntonti, & Kozobolis, 2017; Mahrous, Ciralsky, & Lai, 2018). In fact, the most common complaint is inability to read without glasses, effectively suggesting that suppressing the dominant eye is not easy for all patients despite the high-contrast text presented to the nondominant eye. A related problem arises in unilateral prismatic field expansion in tunnel vision or homonymous hemianopia, where patients should be able to handle the binocular visual confusion caused by seeing two objects at the same visual location if dominance is not too strong; if dominance is strong, the prism has to be fitted on the dominant eye (Ross et al., 2012). These observations suggest that effective determination of ocular dominance and its magnitude is still insufficient for efficacy of the intervention, which should also consider the patient’s ability to selectively suppress one of the eyes or, at least, to ignore the visual information coming from it. Tests that measure this ability are yet to be developed, but they will be a useful companion to ocular dominance tests for prospective clinical intervention.

## Supplemental Material

Supplemental Material1 - Supplemental material for Psychophysical Tests Do Not Identify Ocular Dominance Consistently Click here for additional data file. Supplemental material, Supplemental Material1 for Psychophysical Tests Do Not Identify Ocular Dominance Consistently by Miguel A. García-Pérez and Eli Peli in i-Perception

## Supplemental Material

Supplemental Material2 - Supplemental material for Psychophysical Tests Do Not Identify Ocular Dominance Consistently Click here for additional data file. Supplemental material, Supplemental Material2 for Psychophysical Tests Do Not Identify Ocular Dominance Consistently by Miguel A. García-Pérez and Eli Peli in i-Perception

## Supplemental Material

Supplemental Material3 - Supplemental material for Psychophysical Tests Do Not Identify Ocular Dominance Consistently Click here for additional data file. Supplemental material, Supplemental Material3 for Psychophysical Tests Do Not Identify Ocular Dominance Consistently by Miguel A. García-Pérez and Eli Peli in i-Perception

## Figures and Tables

**Figure B1.**
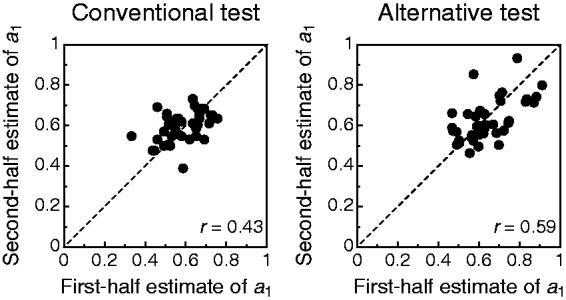
Scatter plots and correlations between split-half estimates of the LE weight *a*_1_ in the conventional (left panel) and alternative (right panel) perceived-phase tests. The dashed diagonal is the identity line. The inset in each panel reports the product-moment correlation.

**Figure C1.**
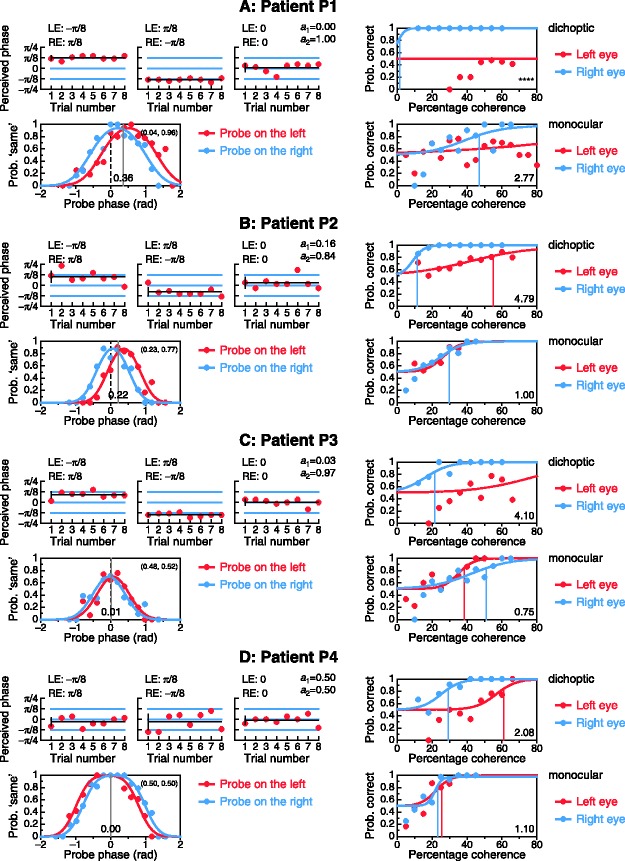
Psychophysical results for four observers with abnormal binocular vision.

## Note

1 Other studies defined the coherence threshold as the 71%-correct point (Li et al., 2011) or the 79%-correct point (e.g., Hess et al., 2007; Li et al., 2010) instead, and this was only because these are the points that presumably provide the average-of-reversals estimates from the adaptive staircases that were used to collect data. The 84%-correct point is instead the target of choice when psychometric functions are fitted to the data because this is the point that corresponds to a *d’* of unity under conventional assumptions. None of the definitions is intrinsically better than any other, and they do not bias the results in any way, but it should be noted that average-of-reversals estimates from conventionally used adaptive staircases are severely flawed (García-Pérez, 1998, 2000, 2011).

## Appendix A

### Fit of Psychometric Functions to Same–Different Data

The software provided by García-Pérez and Alcalá-Quintana (2017) includes multiple options for fitting dual-presentation same–different data. Those used in this study are listed next and readers are referred to the original source for a full description of what these options accomplish. First, we fitted psychometric functions with Type = ‘diff’, which implements the assumption that the psychophysical functions governing perception of phase may differ for probe and standard stimuli (given that the perceived phase of the latter comes from a cyclopean combination that may not end up producing the nominal phase arising from Equation (3) with *a*_1_ = *a*_2_ = 0.5). We also set Standard = 0 to declare the nominal phase of the standard. These two settings ensure that the perceived phase of the standard will come out as the estimated PSE (i.e., the phase of the probe that is perceptually equal to the perceived phase of the standard). Finally, we used the option Model = 5 to fit psychometric functions without lapse/error parameters, after checking that the more general model with all possible lapse/error parameters (Model = 1) did not provide a meaningfully better account of the data. Then, each observer’s data were fitted by a model with four free parameters (namely, β_t_, μ_s_(*x*_s_), δ_1_, and δ_2_; for a description of these parameters, see García-Pérez & Alcalá-Quintana, 2017). For the record, we used an equivalent fortran version of the original software, which runs substantially faster and allows for a more thorough search for the maximum-likelihood solution in parameter space.

### Fit of Psychometric Functions Under the Indecision Model With Different Sensitivities

The indecision model with different sensitivities in dual-presentation tasks posits that different psychophysical functions hold for each presentation location. This model is applicable to data from our alternative coherence-threshold test on evidence that the strength of perceived upward motion varies between hemiretinae within each eye (which defines the two positions in each dual-presentation trial). Additional allowance for differences in perceived strength across eyes implies that separate models are also needed for each eye, requiring four psychophysical functions to cover all conditions. Model development follows as shown in García-Pérez and Alcalá-Quintana (2017) by using the two psychophysical functions that apply to each eye and repeating the development for the two eyes. A sketch is presented next to highlight differences with respect to the case of identical psychophysical functions at both presentation positions.

Let μ_LL_, μ_LR_, μ_RL_, and μ_RR_ be the psychophysical functions describing how the strength of perceived upward motion increases with percentage coherence (the stimulus level *x*, ranging from 0 to 100) when the coherent group is delivered to the left eye in the left hemiretina (LL), to the left eye in the right hemiretina (LR), to the right eye in the left hemiretina (RL), and to the right eye in the right hemiretina (RR), respectively. We assume each of them to be a two-parameter function with the mathematical form in Equation (2) of García-Pérez and Alcalá-Quintana (2017), which would thus contribute eight free parameters (α_LL_, α_LR_, α_RL_, α_RR_, β_LL_, β_LR_, β_RL_, and β_RR_) to the set of model psychometric functions. Yet, it will be seen later that empirical constraints make α_LL_ = α_LR_ = α_RL_ = α_RR_ = 0, leaving only four free parameters as far as the psychophysical functions are concerned. Note also that these psychophysical functions characterize the location where a stimulus is presented, not whether the stimulus is the null or the target (the standard or the test in the terminology of García-Pérez & Alcalá-Quintana, 2017).

Sensory effects are also assumed to be normally distributed (Equation (1) in García-Pérez & Alcalá-Quintana, 2017) with unit variance and mean given by the psychophysical function that applies to the location where the stimulus is presented. The decision variable is thus a normally distributed random variable with variance 2 and mean given by the difference between the applicable psychophysical functions, one of them evaluated at 0 (for the null stimulus) and the other evaluated at *x* (the percentage of coherence in the current trial). By analogy with Equations (3) in García-Pérez and Alcalá-Quintana (2017), these means are μ_LR_(0) − μ_LL_(*x*) when the target is displayed in the LL position, μ_LR_(*x*) − μ_LL_(0) when the target is displayed in the LR position (in the LE model), μ_RR_(0) – μ_RL_(*x*) when the target is displayed in the RL position, and μ_RR_(*x*) − μ_RL_(0) when the target is displayed in the RR position (in the RE model). Two separate models are thus defined (one for each eye), but we will see later that they come together due to common decisional parameters. Also, empirical constraints require μ_LL_(0) = μ_LR_(0) and μ_RL_(0) = μ_RR_(0), which implies that α parameters are inconsequential. As discussed earlier, all α parameters are thus set to zero for convenience so that μ*_ij_*(0) = ln(3) for *i, j* ∈ {L, R}.

Because trials with the two presentation positions in each eye were randomly interwoven and observers could not tell which trial was for what condition, the same boundaries δ_1_ and δ_2_ must hold for all conditions in one-dimensional decision space. For identifiability, the constraint δ_1_ = −δ_2_ was also imposed because differences in performance across presentation positions in each eye are captured by the psychophysical functions under the assumption of different sensitivities to motion. Imposing this constraint does not alter estimated thresholds.

In sum, the basic model psychometric functions include five free parameters across the two eyes and the two presentation positions: a single and unique sensory parameter per eye/position (β_LL_, β_LR_, β_RL_, and β_RR_) and a single decisional parameter (δ_2_) in all cases. The basic model was nevertheless extended to include some error parameters as discussed in García-Pérez and Alcalá-Quintana (2017), mostly because data from some observers seemed to indicate that they guessed about as often as they actually gave “can’t tell” responses. To capture this behavior, error model 6 (see Figure 3 in García-Pérez & Alcalá-Quintana, 2017) was implemented by introducing the error/bias parameters ɛ_U_ and κ_U–F_ (in the notation of the source) at each presentation position.

Custom software for fitting these psychometric functions was written in fortran. Given that data from all conditions contribute to δ_2_, parameter estimates were sought by maximizing the joint likelihood function of the four eye × hemiretina models. Thresholds at 84% correct were then computed from the estimated parameters for each hemiretina in each eye.

## Appendix B

We estimated the reliability of conventional and alternative versions of the perceived-phase test with the split-half method (see, e.g., Furr, 2010). For each observer, the total number of trials administered in each test was first split into two halves of the same size, estimates of ocular weights were then separately computed from the set of trials in each half, and the correlation between split-half estimates was finally computed. The split of data from the conventional test separated data collected in the first and second halves of the session, as this ensured that both halves included equal number of trials for each of the conditions. In contrast, data for the alternative test were collected with multiple randomly interwoven adaptive staircases so that trials at probe phases far away from the standard were used only at the beginning of each staircase. To guarantee that each half of the split used appropriate data for parameter estimation, data were separated by placing all trials belonging in a given staircase into the same split, which ended up producing splits with the same overall numbers of trials given that there was an even number of staircases each of which consisted of the same number of trials. Each split included half of the staircases with the same initial phase and probe location from each session.

Split-half estimates of reliability are lower than test–retest estimates obtained by administering the full test a second time, if only because the precision of measurement increases with number of trials. When raw measures are sum scores, split-half reliability estimates are upgraded to the length (number of trials) of the original test with the Spearman–Brown correction (see, e.g., Kingston & Tiemann, 2010), but this strategy is not applicable when raw measures are obtained otherwise as is the case for ocular weights here. Nevertheless, the impossibility to upgrade our estimates does not preclude a comparative analysis of reliability between tests. Alternatively, the reliability estimates reported here can be strictly interpreted as those of a conventional perceived-phase test with four trials in each offset condition (instead of our original eight trials per offset condition) and an alternative perceived-phase test with 180 trials (90 trials per psychometric function) instead of our original 360 trials (180 per function).

Figure B1 shows scatter plots and correlations that document the reliabilities of the conventional (left panel) and alternative (right panel) versions of the perceived-phase test. Only estimates of the LE ocular weight *a*_1_ are used because estimates of the RE ocular weight *a*_2_ will offer the exact same picture. Note that the underlying true ocular weights are the same in both cases because the same observers are involved and, hence, the smaller variability of estimates obtained with the conventional test is already an indication that true variability was dampened. Consequently, split-half estimates of *a*_1_ are more strongly correlated in the alternative test. We should stress that the necessary precondition of parallelism (equality of means and variances; see García-Pérez, 2013) that warrants interpretation of these correlations as reliability estimates holds here: Parallelism assessed by a Bradley–Blackwood test was rejected neither for the conventional test (*F* = 1.70, *p* = .20) nor for the alternative test (*F* = 0.95, *p* = .40).

Correlations are not as high as one might wish, not even for the alternative perceived-phase test, but there are two obvious reasons for this outcome. First, estimates are computed here for tests that are half the length of those we actually used in our main study; the reliabilities of our full-length tests must be higher than these figures, but there is no simple way in which those can be estimated (other than conducting full-length test-retest studies, of course). Second, estimates are downgraded by range restriction due to the already noted fact that our sample of observers did not happen to include cases of clear RE dominance by these tests (i.e., cases with *a*_1_ ≪ 0.5); had we come across such cases in our sample (on the reasonable assumption that they exist in the population), the bottom-left part of each panel in Figure B1 would have been populated as the top-right part is, and correlations would have been substantially higher. Although corrections have been devised to estimate what the reliability would have been without range restrictions (see, e.g., Alexander, Alliger, & Hanges, 1984; Alexander, Hanges, & Alliger, 1985), we decided against using them because they are not necessary for our purposes but also because of their potential problems (see Johnson, Deary, & Bouchard, 2018).

In any case, these underestimates of reliability do not preclude a comparative analysis that identifies the alternative form of the perceived-phase test as more reliable than the conventional form. It should also be stressed that this only says that the alternative test provides more dependable measures of whatever these tests measure, be it ocular dominance or something else.

## Appendix C

We recruited four observers with abnormal binocular vision and measured their performance in the conventional and alternative forms of the perceived-phase test and in the conventional form of the coherence-threshold test, and we also measured their monocular psychometric functions in the coherent-motion detection task. Except for one of the observers, their vision was known to suffer from strong suppression of one of the eyes, and this was confirmed in the eye examination that preceded data collection. Observers’ clinical details are given in Table C1. Although suppression in abnormal binocular vision is not equivalent to strong ocular dominance in normal binocular vision (i.e., there is not a true and fair competition between the eyes in the former case), the pattern of results displayed by these observers may shed some light on the interpretability of the results of psychophysical tests as evidence of ocular dominance.

**Table C1.** Clinical Characteristics of Observers.

ID	Diagnosis	BCVA OD (LogMAR)	BCVA OS (LogMAR)	Stereopsis	Randot Suppression	Worth 4-Dot	Bagolini	Rx SE OD	Rx SE OS	Gender	Age
P1	L exotropia^a^	0.30	1.44	None	R|	diplopia	RD	−1.50	−1.50	Male	23
P2	L esotropia^b^	−0.04	0.40	None^c^	R|	RD	RD	+5.63	+9.50	Female	55
P3	L esotropia^d^	0.00	0.10	None^e^	R|L	diplopia	RD	+0.13	+4.63	Male	67
P4	L exotropia^f^	0.00	0.00	20 arc sec	R+L	ND	ND	0.00	0.00	Male	42

*Note*. BCVA = best corrected visual acuity; SE = spherical equivalent; OD = right eye; OS = left eye; R = right; L = left; RD = right dominance; ND = no dominance.

a Constant exotropia with left amblyopia. Postsurgery for constant esotropia at age 14 years.

b Accommodative intermittent esotropia with amblyopia treated with occlusion therapy from age 5 years.

c None on Randot Stereotest; recently was able to appreciate stereo in a wide screen 3D movie.

d Accommodative esotropia treated by Visual Training at childhood (ages 5–6 years).

e None on Randot Stereotest; was able to see large disparity with large stereo imagery, even random dot stereograms.

f Intermittent. Postsurgery for accommodative esotropia at age 4 or 5 years.

It is thus instructive to discuss what results are expected under suppression of one eye. In either form of the perceived-phase test, performance would be determined only by the nonsuppressed eye, which would render severely imbalanced estimates of ocular weights (i.e., either *a*_1_ ≈ 1 or *a*_2_ ≈ 1 according to which eye is suppressed). If performance in the coherence-threshold test is analogously determined by input from the nonsuppressed eye, then observers would be 100% correct when any number of target dots are presented to it, since the noise dots presented to the suppressed eye are not seen; similarly, performance would be at the 50% chance level when target dots are presented to the suppressed eye, as the observer only sees noise dots that move neither to the left nor to the right. What monocular coherence thresholds should be like is harder to anticipate because here noise and target dots are presented to the same eye while the other eye remains unstimulated. In these conditions, monocular performance with the nonsuppressed eye would not be perfect because target and noise dots are always visible, but whether this performance is different from monocular performance with the suppressed eye is uncertain. If suppression were equivalent to visual signals not being transmitted out of the suppressed eye, monocular performance with the suppressed eye would be at chance at all coherence levels because of an effective lack of visual input; if suppression were instead equivalent to visual signals from the suppressed eye losing to visual signals from the nonsuppressed eye at some point up the visual pathway, monocular performance with the suppressed eye might be similar to performance with the nonsuppressed eye due to lack of competing signals.

Results are shown in Figure C1. Two of the observers show unmistakable signs of suppression in both forms of the perceived-phase test (Figure C1(a) and (b), left side), with ocular weights that consistently reveal a null or almost null LE contribution, in agreement with the clinical condition of these observers (see Table C1). A third observer (Figure C1(c), left side) also displays virtually absent LE contribution in the conventional perceived-phase test, which contrasts with a balanced contribution from both eyes in the alternative form of the test. The discrepancy between the two forms of the perceived-phase test can also be understood from the clinical condition of this observer, who has some stereo vision with large imagery. Thus, binocular function does not operate in the narrow field and central viewing associated with the conventional form of the test but it does in the broad field with peripheral viewing associated with the alternative form. The fourth observer, on the other hand, does not show any sign of imbalanced ocular contributions in either form of the perceived-phase test (Figure C1(d), left side), consistent with the fact that the clinical evaluation of this observer does not show any sign of suppression (see Table C1). Thus, these results are in good agreement with expectations based on the known visual function of these observers and confirm that the perceived-phase test reflects the outcome of a competition between the eyes.

In contrast, the results of the coherence-threshold test and the monocular psychometric functions (Figure C1, right side) are more diverse across observers and harder to interpret. The results for the first observer (Figure C1(a)) suggest a dysfunction comparable to functional absence of the suppressed left eye: Dichoptically, performance is perfect at all coherence levels when target dots are presented to the nonsuppressed eye, and it is at chance when target dots are presented to the suppressed eye; monocularly, performance with the suppressed eye is also essentially at chance, and it is very poor with the nonsuppressed eye (but note that there is no reason to expect good or bad monocular performance with the nonsuppressed eye). In contrast, the next observer (Figure C1(b)) displays similar monocular performance with both eyes, which transforms into remarkably better dichoptic performance with the nonsuppressed eye, as if both eyes were functionally similar when tested in isolation. On the other hand, the third observer (Figure C1(c)) also shows analogously poor monocular performance that is nevertheless slightly better with the suppressed left eye, whereas dichoptic performance with the suppressed left eye is essentially at chance level. Finally, the fourth observer (Figure C1(d)), who did not show any clinically measurable ocular suppression (see Table C1) and showed equal ocular contributions by the perceived-phase tests (Figure C1(d), left side), nevertheless displayed clear signs of LE impairment in the coherence-threshold test accompanied with good and nearly identical monocular performance with either eye. Then, ocular suppression (observers (a) to (c) in Figure C1) is confounded with an unidentified factor (observer (d) in Figure C1) in the conventional coherence-threshold test, which appears unsuited for measuring ocular dominance.
