# Supplementary material for: Psychophysical Tests Do Not Identify Ocular Dominance Consistently
Source: Iperception. 2019 Apr 29;10(2):2041669519841397. doi: 10.1177/2041669519841397 (PMC6492369; doi:10.1177/2041669519841397)
Supplement: Supplemental Material1 - Supplemental material for Psychophysical Tests Do Not Identify Ocular Dominance Consistently [file Supplemental_Material1.pdf]

Appendix A

A1. Fit of psychometric functions to same–different data

The software provided by García-Pérez and Alcalá-Quintana (2017) includes multiple options for fitting dual-presentation same–different data. Those used in this study are listed next and readers are referred to the original source for a full description of what these options accomplish. First, we fitted psychometric functions with `Type = 'diff'`, which implements the assumption that the psychophysical functions governing perception of phase may differ for probe and standard stimuli (given that the perceived phase of the latter comes from a cyclopean combination that may not end up producing the nominal phase arising from Eq. 3 with $a_1 = a_2 = 0.5$). We also set `Standard = 0` to declare the nominal phase of the standard. These two settings ensure that the perceived phase of the standard will come out as the estimated PSE (i.e., the phase of the probe that is perceptually equal to the perceived phase of the standard). Finally, we used the option `Model = 5` to fit psychometric functions without lapse/error parameters, after checking that the more general model with all possible lapse/error parameters (`Model = 1`) did not provide a meaningfully better account of the data. Then, each observer's data were fitted by a model with four free parameters (namely, β_t, $\mu_s(x_s)$, δ_1, and δ_2; for a description of these parameters, see García-Pérez and Alcalá-Quintana, 2017). For the record, we used an equivalent FORTRAN version of the original software, which runs substantially faster and allows for a more thorough search for the maximum-likelihood solution in parameter space.

A2. Fit of psychometric functions under the indecision model with different sensitivities

The indecision model with different sensitivities in dual-presentation tasks posits that different psychophysical functions hold for each presentation location. This model is applicable to data from our alternative coherence-threshold test on evidence that the strength of perceived upward motion varies between hemiretinae within each eye (which defines the two positions in each dual-presentation trial). Additional allowance for differences in perceived strength across eyes implies that separate models are also needed for each eye, requiring four psychophysical functions to cover all conditions. Model development follows as shown in García-Pérez and Alcalá-Quintana (2017) by using the two psychophysical functions that apply to each eye and repeating the development for the two eyes. A sketch is presented next to highlight differences with respect to the case of identical psychophysical functions at both presentation positions.

Let μ_{LL}, μ_{LR}, μ_{RL}, and μ_{RR} be the psychophysical functions describing how the strength of perceived upward motion increases with percentage coherence (the stimulus level x, ranging from 0 to 100) when the coherent group is delivered to the left eye in the left hemiretina (LL), to the left eye in the right hemiretina (LR), to the right eye in the left hemiretina (RL), and to the right eye in the right hemiretina (RR), respectively. We assume each of them to be a two-parameter function with the mathematical form in equation 2 of García-Pérez and Alcalá-Quintana (2017), which would thus contribute eight free parameters (α_{LL}, α_{LR}, α_{RL}, α_{RR}, β_{LL}, β_{LR}, β_{RL}, and β_{RR}) to the set of model psychometric functions. Yet, it will be seen later that empirical constraints make $\alpha_{LL} = \alpha_{LR} = \alpha_{RL} = \alpha_{RR} = 0$, leaving only four free parameters as far as the psychophysical functions are concerned. Note also that these psychophysical functions characterize the location where a stimulus is presented, not whether the stimulus is the null or the target (the standard or the test in the terminology of García-Pérez and Alcalá-Quintana, 2017).

Sensory effects are also assumed to be normally distributed (equation 1 in García-Pérez and Alcalá-Quintana, 2017) with unit variance and mean given by the psychophysical function that applies to the location where the stimulus is presented. The decision variable is thus a normally-distributed random variable with variance 2 and mean given by the difference between the applicable psychophysical functions, one of them evaluated at 0 (for the null stimulus) and the other evaluated at x (the percentage of coherence in the current trial). By analogy with equations 3 in García-Pérez and Alcalá-Quintana (2017), these means are $\mu_{LR}(0) - \mu_{LL}(x)$ when the target is displayed in the LL position, $\mu_{LR}(x) - \mu_{LL}(0)$ when the target is displayed in the LR position (in the LE model), $\mu_{RR}(0) - \mu_{RL}(x)$ when the target is displayed in the RL position, and $\mu_{RR}(x) - \mu_{RL}(0)$ when the target is displayed in the RR position (in the RE model). Two separate models are thus defined (one for each eye) but we will see later that they come together due to common decisional parameters. Also, empirical constraints require $\mu_{LL}(0) = \mu_{LR}(0)$ and $\mu_{RL}(0) = \mu_{RR}(0)$, which implies that α parameters are inconsequential. As discussed earlier, all α parameters are thus set to zero for convenience so that $\mu_{ij}(0) = \ln(3)$ for $i,j \in \{L, R\}$.

Because trials with the two presentation positions in each eye were randomly interwoven and observers could not tell which trial was for what condition, the same boundaries δ_1 and δ_2 must hold for all conditions in one-dimensional decision space. For identifiability, the constraint $\delta_1 = -\delta_2$ was also imposed because differences in performance across presentation positions in each eye are captured by the psychophysical functions under the assumption of different sensitivities

to motion. Imposing this constraint does not alter estimated thresholds.

In sum, the basic model psychometric functions include five free parameters across the two eyes and the two presentation positions: A single and unique sensory parameter per eye/position (β_{LL}, β_{LR}, β_{RL}, and β_{RR}) and a single decisional parameter (δ_2) in all cases. The basic model was nevertheless extended to include some error parameters as discussed in García-Pérez and Alcalá-Quintana (2017), mostly because data from some observers seemed to indicate that they guessed about as often as they actually gave "can't tell" responses. To capture this behavior, error model 6 (see figure 3 in García-Pérez and Alcalá-Quintana, 2017) was implemented by introducing the error/bias parameters ε_U and κ_{U-F} (in the notation of the source) at each presentation position.

Custom software for fitting these psychometric functions was written in FORTRAN. Given that data from all conditions contribute to δ_2, parameter estimates were sought by maximizing the joint likelihood function of the four eye×hemiretina models. Thresholds at 84% correct were then computed from the estimated parameters for each hemiretina in each eye.

Appendix B

We estimated the reliability of conventional and alternative versions of the perceived-phase test with the split-half method (see, e.g., Furr, 2010). For each observer, the total number of trials administered in each test was first split into two halves of the same size, estimates of ocular weights were then separately computed from the set of trials in each half, and the correlation between split-half estimates was finally computed. The split of data from the conventional test separated data collected in the first and second halves of the session, as this ensured that both halves included equal number of trials for each of the conditions. In contrast, data for the alternative test were collected with multiple randomly interwoven adaptive staircases so that trials at probe phases far away from the standard were used only at the beginning of each staircase. To guarantee that each half of the split used appropriate data for parameter estimation, data were separated by placing all trials belonging in a given staircase into the same split, which ended up producing splits with the same overall numbers of trials given that there was an even number of staircases each of which consisted of the same number of trials. Each split included half of the staircases with the same initial phase and probe location from each session.

Split-half estimates of reliability are lower than test-retest estimates obtained by administering the full test a second time, if only because the precision of measurement increases with number

of trials. When raw measures are sum scores, split-half reliability estimates are upgraded to the length (number of trials) of the original test with the Spearman–Brown correction (see, e.g., Kingston & Tiemann, 2010), but this strategy is not applicable when raw measures are obtained otherwise as is the case for ocular weights here. Nevertheless, the impossibility to upgrade our estimates does not preclude a comparative analysis of reliability between tests. Alternatively, the reliability estimates reported here can be strictly interpreted as those of a conventional perceived-phase test with four trials in each offset condition (instead of our original eight trials per offset condition) and an alternative perceived-phase test with 180 trials (90 trials per psychometric function) instead of our original 360 trials (180 per function).

Figure B1 shows scatter plots and correlations that document the reliabilities of the conventional (left panel) and alternative (right panel) versions of the perceived-phase test. Only estimates of the LE ocular weight a_1 are used because estimates of the RE ocular weight a_2 will offer the exact same picture. Note that the underlying true ocular weights are the same in both cases because the same observers are involved and, hence, the smaller variability of estimates obtained with the conventional test is already an indication that true variability was dampened. Consequently, split-half estimates of a_1 are more strongly correlated in the alternative test. We should stress that the necessary pre-condition of parallelism (equality of means and variances; see García-Pérez, 2013) that warrants interpretation of these correlations as reliability estimates holds here: Parallelism assessed by a Bradley–Blackwood test was rejected neither for the conventional test ($F = 1.70$, $p = .20$) nor for the alternative test ($F = 0.95$, $p = .40$).

Correlations are not as high as one might wish, not even for the alternative perceived-phase test, but there are two obvious reasons for this outcome. First, estimates are computed here for tests that are half the length of those we actually used in our main study; the reliabilities of our full-length tests must be higher than these figures, but there is no simple way in which those can be estimated (other than conducting full-length test-retest studies, of course). Second, estimates are downgraded by range restriction due to the already noted fact that our sample of observers did not happen to include cases of clear RE dominance by these tests (i.e., cases with $a_1 \ll 0.5$); had we come across such cases in our sample (on the reasonable assumption that they exist in the population), the bottom-left part of each panel in Fig. B1 would have been populated as the top-right part is, and correlations would have been substantially higher. Although corrections have been devised to estimate what the reliability would have been without range restrictions (see,

e.g., Alexander, Alliger, & Hanges, 1984; Alexander, Hanges, & Alliger, 1985), we decided against using them because they are not necessary for our purposes but also because of their potential problems (see Johnson, Deary, & Bouchard, 2018).

In any case, these underestimates of reliability do not preclude a comparative analysis that identifies the alternative form of the perceived-phase test as more reliable than the conventional form. It should also be stressed that this only says that the alternative test provides more dependable measures of whatever these tests measure, be it ocular dominance or something else.

Appendix C

We recruited four observers with abnormal binocular vision and measured their performance in the conventional and alternative forms of the perceived-phase test and in the conventional form of the coherence-threshold test, and we also measured their monocular psychometric functions in the coherent-motion detection task. Except for one of the observers, their vision was known to suffer from strong suppression of one of the eyes and this was confirmed in the eye exam that preceded data collection. Observers' clinical details are given in Table C1. Although suppression in abnormal binocular vision is not equivalent to strong ocular dominance in normal binocular vision (i.e., there is not a true and fair competition between the eyes in the former case), the pattern of results displayed by these observers may shed some light on the interpretability of the results of psychophysical tests as evidence of ocular dominance.

Table C1. Clinical characteristics of observers.

ID	Diagnosis	BCVA OD (LogMAR)	BCVA OS (LogMAR)	Stereopsis	Randot Suppression	Worth 4-Dot	Bagolini	Rx SE OD	Rx SE OS	Gender	Age
P1	L exotropia[1]	0.30	1.44	none	R|	diplopia	RD	−1.50	−1.50	male	23
P2	L esotropia[2]	−0.04	0.40	none[a]	R|	RD	RD	+5.63	+9.50	female	55
P3	L esotropia[3]	0.00	0.10	none[b]	R|L	diplopia	RD	+0.13	+4.63	male	67
P4	L exotropia[4]	0.00	0.00	20 arc sec	R+L	ND	ND	0.00	0.00	male	42

BCVA: Best corrected visual acuity; SE: Spherical equivalent; OD: Right eye; OS: Left eye; R: Right; L: Left; RD: Right dominance; ND: No dominance

[1] Constant exotropia with left amblyopia. Post-surgery for constant esotropia at age 14

[2] Accommodative intermittent esotropia with amblyopia treated with occlusion therapy from age 5

[3] Accommodative esotropia treated by Visual Training at childhood (ages 5–6)

[4] Intermittent. Post-surgery for accommodative esotropia at age 4 or 5

[a] None on Randot Stereotest. Recently was able to appreciate stereo in a wide screen 3D movie

[b] None on Randot Stereotest. Was able to see large disparity with large stereo imagery, even random dot stereograms

It is thus instructive to discuss what results are expected under suppression of one eye. In either form of the perceived-phase test, performance would be determined only by the non-suppressed eye, which would render severely imbalanced estimates of ocular weights (i.e., either

$a_1 \approx 1$ or $a_2 \approx 1$ according to which eye is suppressed). If performance in the coherence-threshold test is analogously determined by input from the non-suppressed eye, then observers would be 100% correct when any number of target dots are presented to it, since the noise dots presented to the suppressed eye are not seen; similarly, performance would be at the 50% chance level when target dots are presented to the suppressed eye, as the observer only sees noise dots that move neither to the left nor to the right. What monocular coherence thresholds should be like is harder to anticipate because here noise and target dots are presented to the same eye while the other eye remains unstimulated. In these conditions, monocular performance with the non-suppressed eye would not be perfect because target and noise dots are always visible, but whether this performance is different from monocular performance with the suppressed eye is uncertain. If suppression were equivalent to visual signals not being transmitted out of the suppressed eye, monocular performance with the suppressed eye would be at chance at all coherence levels because of an effective lack of visual input; if suppression were instead equivalent to visual signals from the suppressed eye losing to visual signals from the non-suppressed eye at some point up the visual pathway, monocular performance with the suppressed eye might be similar to performance with the non-suppressed eye due to lack of competing signals.

Results are shown in Fig. C1. Two of the observers show unmistakable signs of suppression in both forms of the perceived-phase test (left side of parts A and B in Fig. C1), with ocular weights that consistently reveal a null or almost null LE contribution, in agreement with the clinical condition of these observers (see Table C1). A third observer (left side of part C in Fig. C1) also displays virtually absent LE contribution in the conventional perceived-phase test, which contrasts with a balanced contribution from both eyes in the alternative form of the test. The discrepancy between the two forms of the perceived-phase test can also be understood from the clinical condition of this observer, who has some stereo vision with large imagery. Thus, binocular function does not operate in the narrow-field and central viewing associated with the conventional form of the test but it does in the broad-field with peripheral viewing associated with the alternative form. The fourth observer, on the other hand, does not show any sign of imbalanced ocular contributions in either form of the perceived-phase test (left side of part D in Fig. C1), consistent with the fact that the clinical evaluation of this observer does not show any sign of suppression (see Table C1). Thus, these results are in good agreement with expectations

based on the known visual function of these observers and confirm that the perceived-phase test reflects the outcome of a competition between the eyes.

In contrast, the results of the coherence-threshold test and the monocular psychometric functions (right side of Fig. C1) are more diverse across observers and harder to interpret. The results for the first observer (part A in Fig. C1) suggest a dysfunction comparable to functional absence of the suppressed left eye: Dichoptically, performance is perfect at all coherence levels when target dots are presented to the non-suppressed eye and it is at chance when target dots are presented to the suppressed eye; monocularly, performance with the suppressed eye is also essentially at chance and it is very poor with the non-suppressed eye (but note that there is no reason to expect good or bad monocular performance with the non-suppressed eye). In contrast, the next observer (part B in Fig. C1) displays similar monocular performance with both eyes, which transforms into remarkably better dichoptic performance with the non-suppressed eye, as if both eyes were functionally similar when tested in isolation. On the other hand, the third observer (part C in Fig. C1) also shows analogously poor monocular performance that is nevertheless slightly better with the suppressed left eye whereas dichoptic performance with the suppressed left eye is essentially at chance level. Finally, the fourth observer (part D in Fig. C1), who did not show any clinically measurable ocular suppression (see Table C1) and showed equal ocular contributions by the perceived-phase tests (left side of part D in Fig. C1), nevertheless displayed clear signs of LE impairment in the coherence-threshold test accompanied with good and nearly identical monocular performance with either eye. Then, ocular suppression (observers A–C in Fig. C1) is confounded with an unidentified factor (observer D in Fig. C1) in the conventional coherence-threshold test, which appears unsuited for measuring ocular dominance.
