# Supplementary figures and images for: Psychophysical Tests Do Not Identify Ocular Dominance Consistently
Source: Iperception. 2019 Apr 29;10(2):2041669519841397. doi: 10.1177/2041669519841397 (PMC6492369; doi:10.1177/2041669519841397)

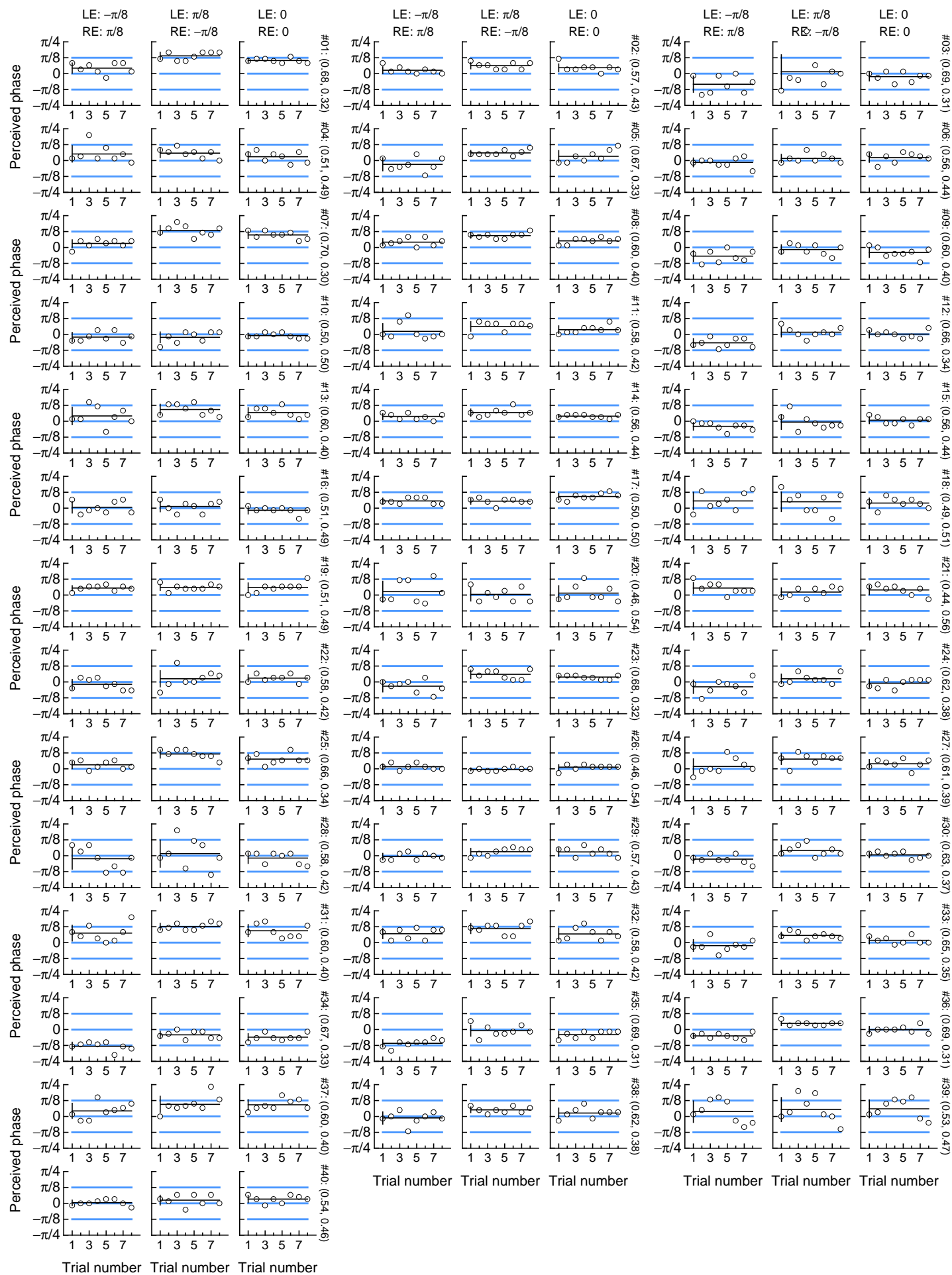

Supplement: Supplemental Material2 - Supplemental material for Psychophysical Tests Do Not Identify Ocular Dominance Consistently [file Supplemental_Material2.pdf]

## Left eye

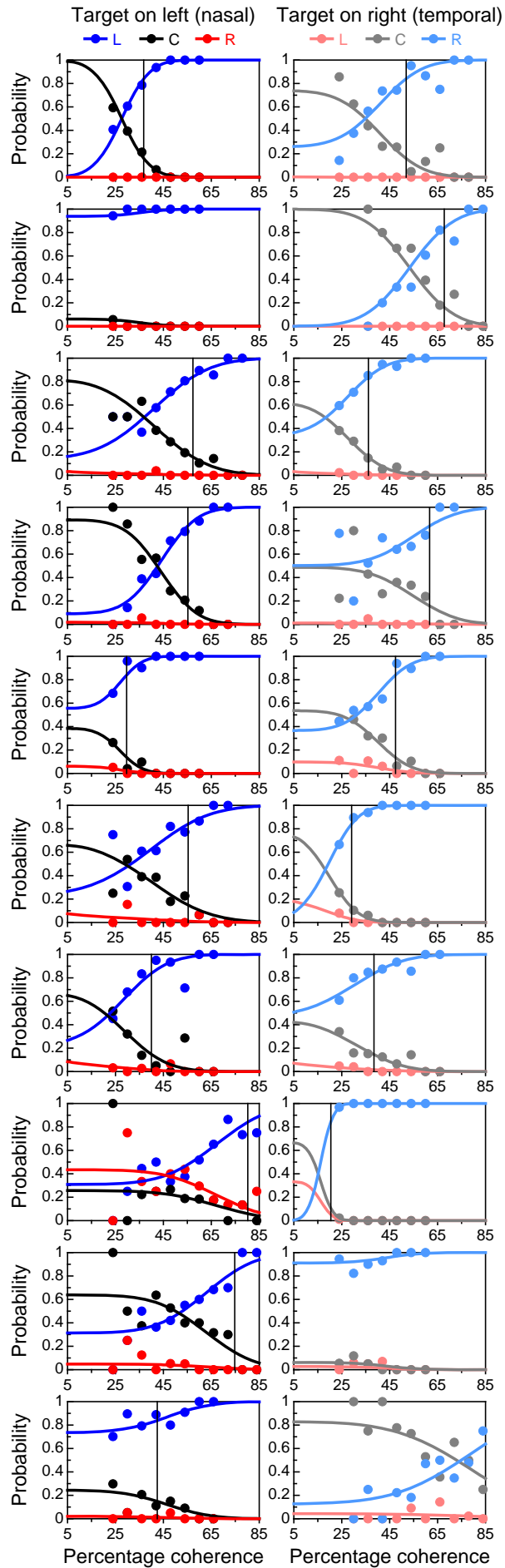

## Right eye

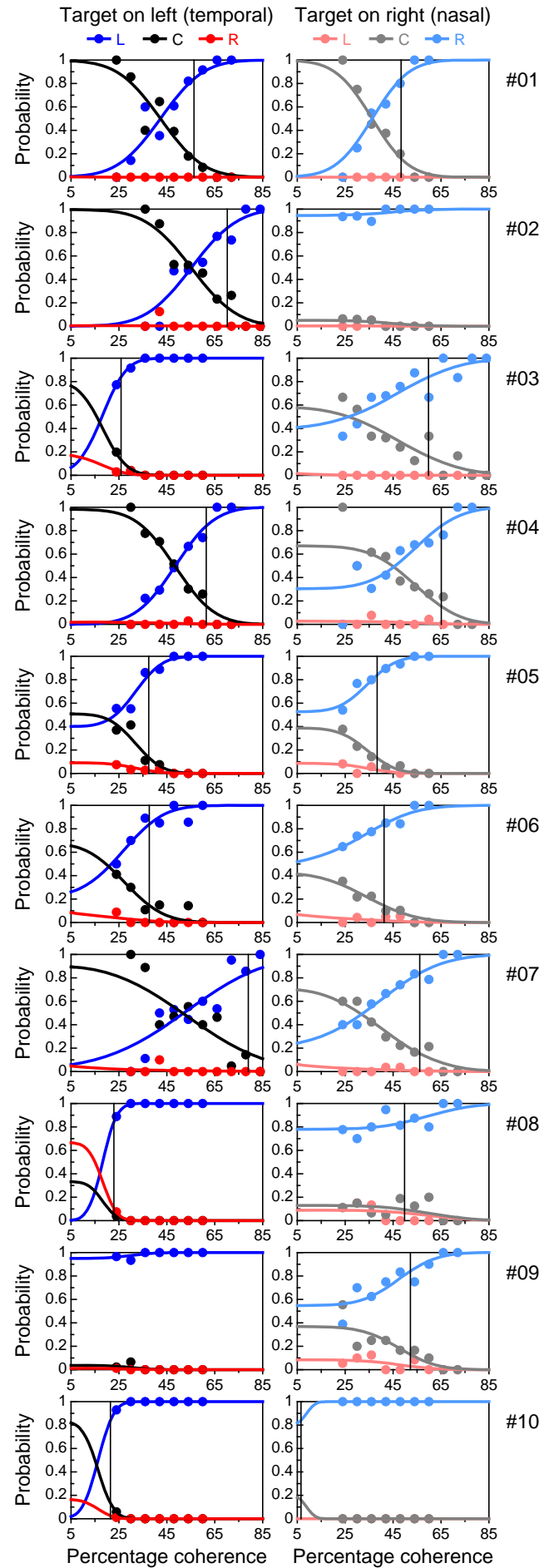

## Left eye

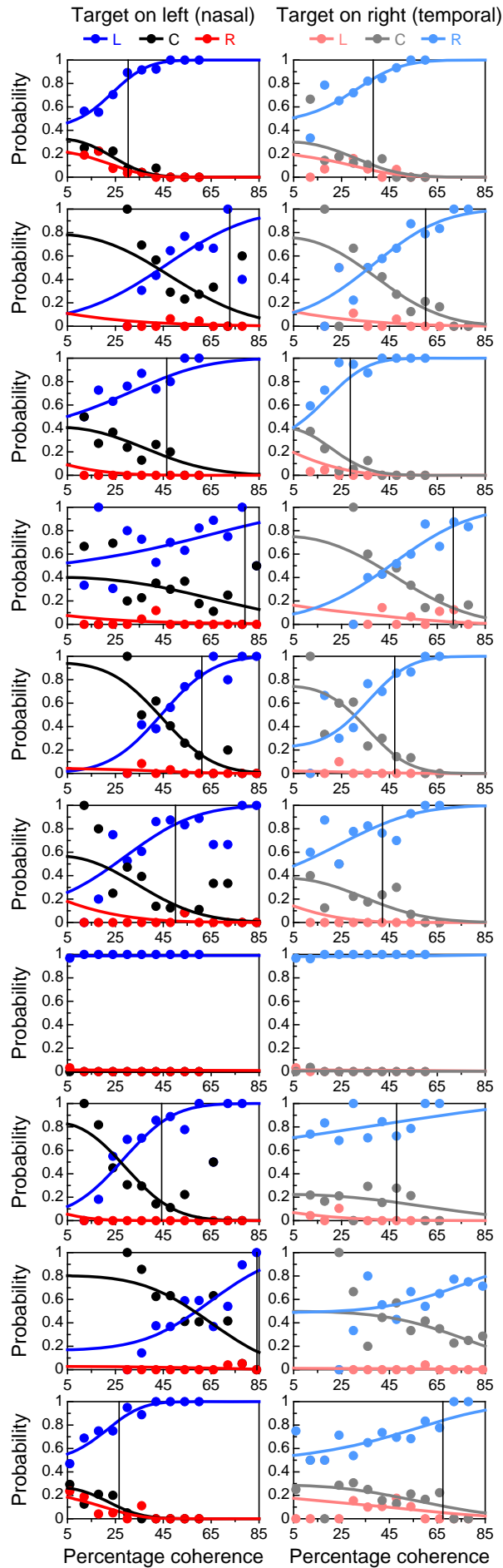

## Right eye

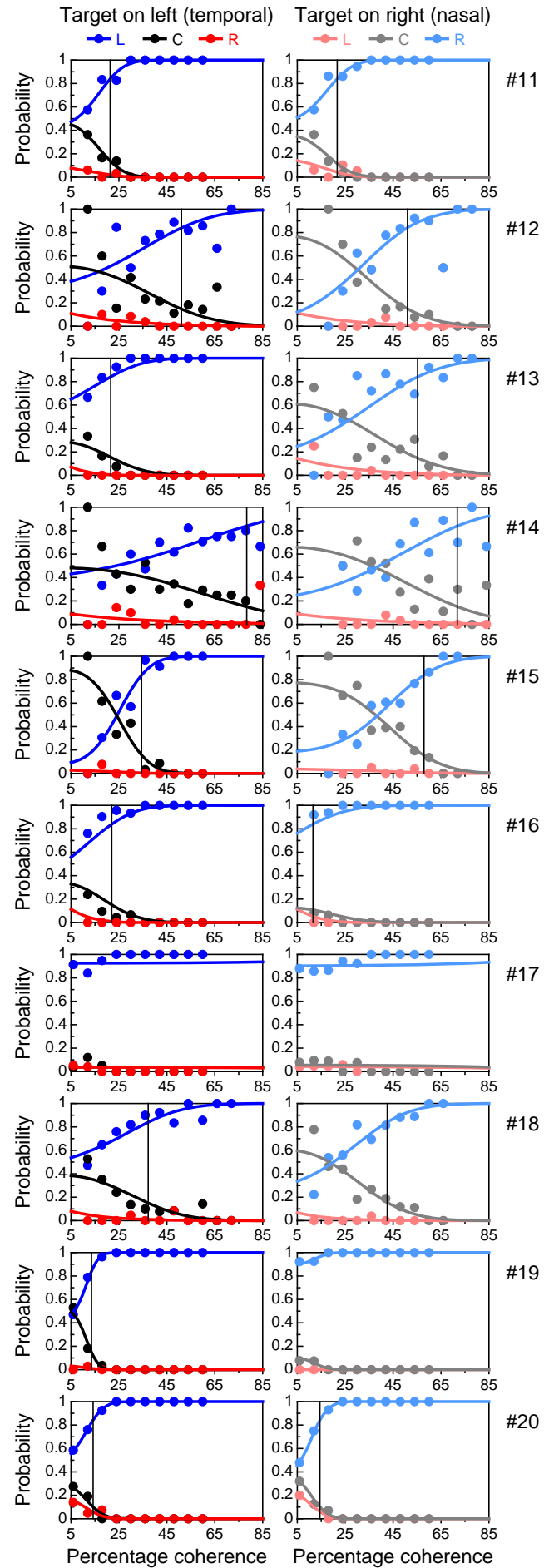

## Left eye

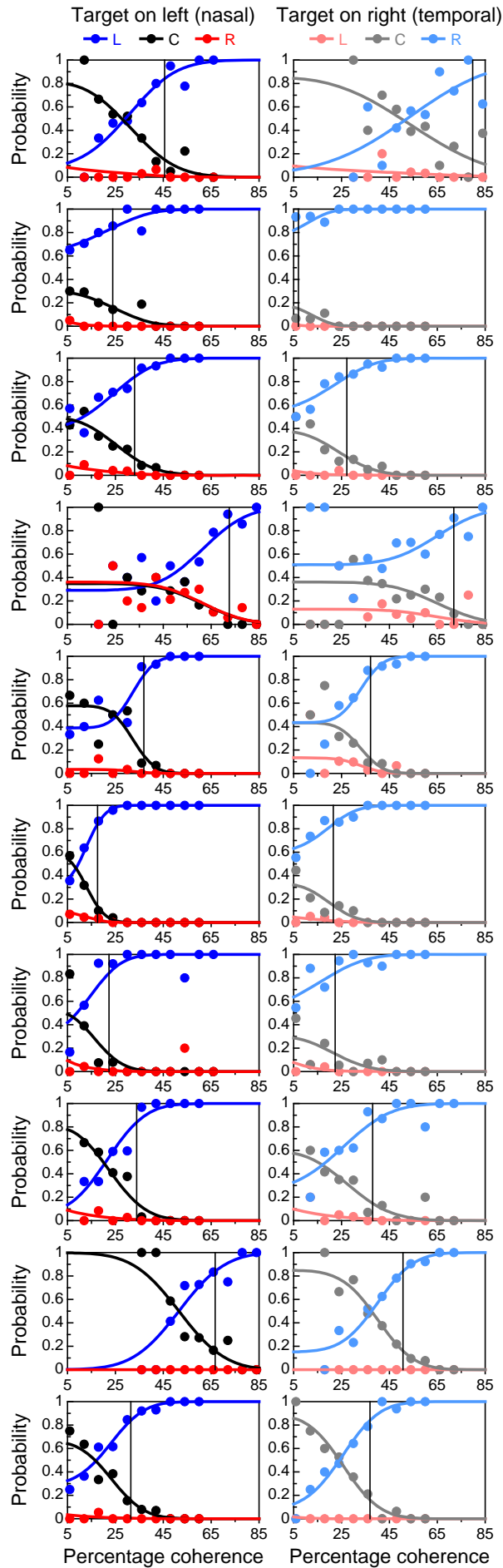

## Right eye

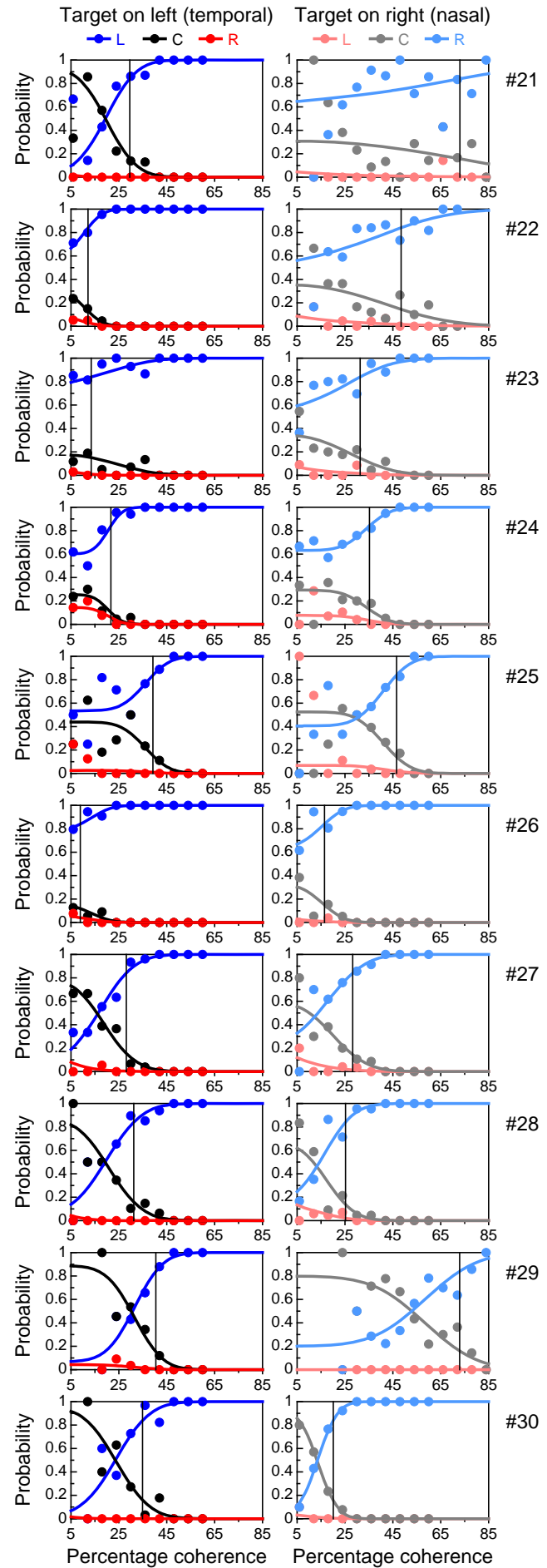

## Left eye

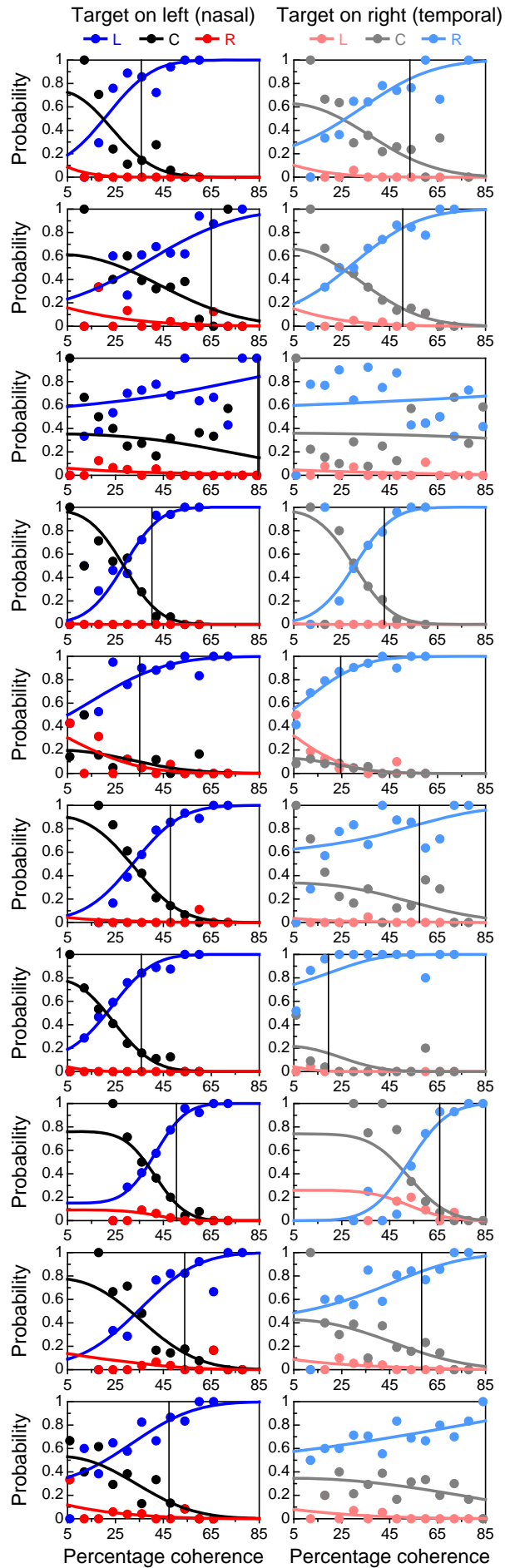

## Right eye

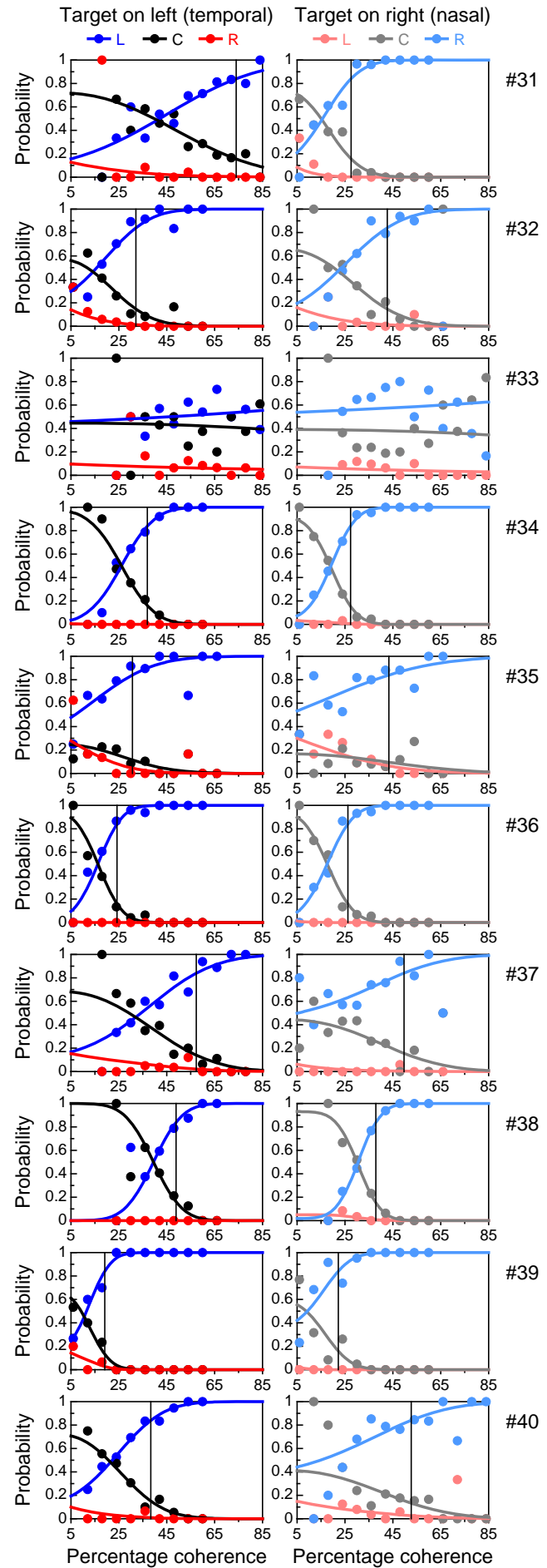

Supplement: Supplemental Material3 - Supplemental material for Psychophysical Tests Do Not Identify Ocular Dominance Consistently [file Supplemental_Material3.pdf]
